# Dysfunctional DNA repair pathway via defective *FANCD2* gene engenders multifarious exomic and transcriptomic effects in Fanconi anemia

**DOI:** 10.1002/mgg3.502

**Published:** 2018-11-18

**Authors:** Karthik Raja Velmurugan, Pawel Michalak, Lin Kang, Natalie C. Fonville, Harold R. Garner

**Affiliations:** ^1^ Primary Care Research Network and the Center for Bioinformatics and Genetics Edward Via College of Osteopathic Medicine Blacksburg Virginia; ^2^ Center for One Health Research Virginia‐Maryland College of Veterinary Medicine Blacksburg Virginia; ^3^ Institute of Evolution University of Haifa Haifa Israel; ^4^ Riverside Law LLP Glenhardie Corporate Center Wayne Pennsylvania; ^5^ The Gibbs Cancer Center and Research Institute Spartanburg South Carolina; ^6^ Present address: Bioinformatics Group, Channing Division of Network Medicine Brigham and Women's Hospital Boston Massachusetts

**Keywords:** DNA repair, FANCD2, Fanconi anemia, mutation accumulation, next‐generation sequencing, transcriptomic analysis, variant analysis

## Abstract

**Background:**

Fanconi anemia (FA) affects only one in 130,000 births, but has severe and diverse clinical consequences. It has been theorized that defects in the FA DNA cross‐link repair complex lead to a spectrum of variants that are responsible for those diverse clinical phenotypes.

**Methods:**

Using NextGen sequencing, we show that a clinically derived FA cell line had accumulated numerous genetic variants, including high‐impact mutations, such as deletion of start codons, introduction of premature stop codons, missense mutations, and INDELs.

**Results:**

About 65% of SNPs and 55% of INDELs were found to be commonly present in both the FA dysfunctional and retrovirally corrected cell lines, showing their common origin. The number of INDELs, but not SNPs, is decreased in *FANCD2*‐corrected samples, suggesting that FANCD2 deficiency preferentially promotes the origin of INDELs. These genetic modifications had a considerable effect on the transcriptome, with statistically significant changes in the expression of 270 genes. These genetic and transcriptomic variants significantly impacted pathways and molecular functions, spanning a diverse spectrum of disease phenotypes/symptoms, consistent with the disease diversity seen in FA patients.

**Conclusion:**

These results underscore the consequences of defects in the DNA cross‐link repair mechanism and indicate that accumulating diverse mutations from individual parent cells may make it difficult to anticipate the longitudinal clinical behavior of emerging disease states in an individual with FA.

## 1 BACKGROUND

Fanconi anemia (FA) is a congenital disorder, characterized by bone marrow failure, infection susceptibility, and a predisposition to cancer, primarily acute myeloid leukemia (AML) and squamous cell carcinoma (Bogliolo & Surralles, 2015; Kutler et al., 2003; Mathew, 2006; Nalepa & Clapp, 2018). FA cells have been found to be enriched with chromosomal aberrations that occur due to DNA cross‐linking left uncorrected by a dysfunctional DNA repair mechanism, referred to as the (canonical) FA pathway (Che, Zhang, Nepal, Han, & Fei, 2018; Deans & West, 2011; Donahue & Campbell, 2004; Mace‐Aime, Couve, Khassenov, Rosselli, & Saparbaev, 2010; Nalepa & Clapp, 2018). To diagnose FA, hypersensitivity to DNA cross‐linking agents, such as mitomycin C (MMC), diepoxybutane (DEB), and cisplatin, is usually used (Auerbach, 2009; Meyer, Neitzel, & Tönnies, 2012; Taniguchi & D'Andrea, 2006). DNA cross‐linking agents have also been used as cytotoxic drugs to disrupt cell division in cancer, but the cell’s response to such a treatment has not been entirely understood (Brulikova, Hlavac, & Hradil, 2012). Recruiting DNA repair proteins to the interstrand cross‐links (ICLs) is known to be the primary function of the FA pathway, and recent studies show the involvement of the FA pathway in maintaining general genome stability (Michl, Zimmer, & Tarsounas, 2016; Nalepa & Clapp, 2018).

At least 22 genes have been identified to be coding for the FA pathway, classified into complementation groups (Che et al., 2018; Cheung & Taniguchi, 2017; Nalepa & Clapp, 2018). All these complementation groups have been identified as biallelic germline mutations that cause FA, with the exception of FANCB and FANCR (Rad51) (Ameziane et al., 2015; Meetei et al., 2004). One of the genes, originally named *FANCD1*, turned out to be *BRCA2* (OMIM #600185) in which mono‐allelic mutations cause susceptibility to breast and other cancers, while biallelic mutations lead to FA (Howlett et al., 2002; Mathew, 2006). These genes in FA patients exhibit a wide spectrum of mutations, including deletions, frameshifts, stop codons, splice‐site mutations, and missense mutations (Joenje & Patel, 2001). The canonical FA signaling pathway is often divided into three parts (Che et al., 2018). Part I consists of nine known FA proteins (FANCA, B, C, E, F, G, L, M, T, and possibly I) together with FAAPs (FAAP 20/24/100 and MHF1/2) and other known proteins, responsible for the activity of ubiquitin E3 ligase for the monoubiquitination of FANCD2 (OMIM #613984) and FANCI (OMIM #611360). Part II, the FA ID complex, comprises *FANCD2* and its paralog *FANCI* and forms a central axis to connect and orchestrate the entire FA signaling pathway (Che et al., 2018). Part III, the functional units downstream of part II, contains DNA repair proteins that act together following the activation/monoubiquitination of FANCD2/FANCI. Despite the considerable progress in dissecting the genetic and molecular mechanisms of FA signaling, the pathway, especially its parts I and III, likely includes many other genes that have yet to be identified (Che et al., 2018).

In this work, we undertake an effort to understand the genomewide and transcriptome‐wide downstream effects of a dysfunctional *FANCD2* gene in PD20 cell cultures. We hypothesize that one of the downstream consequences of FA‐induced ICL is disrupted transcription, which can lead to altered transcriptional products. It has been shown that transcriptional products can be altered in cancer cells (Silva et al., 2010). A significant level of transcriptome instability has been shown to always be present in cells, and several stabilizing mechanisms are known to be in place to respond to them (Radhakrishnan & Green, [Link]). However, in the absence of a functional FA pathway, we propose that an increased level of unrepaired ICLs can overwhelm the transcriptome stability‐maintaining mechanisms and lead to altered transcriptional products, in which case, consequent impairments of cellular function are imminent. While the ICL repair function of the FA pathway is well known, there are studies that suggest that the FA pathway is involved in the general upkeep of genomic stability (Michl et al., 2016). Additionally, microsatellite instability (MIS) has been linked to dysfunctional mismatch repair and recent studies show the contribution of the cross‐link repair (FA) pathway to microsatellite instability (Concannon & Lahue, 2014; Hubert, Lin, Dion, & Wilson, 2011; Lin, Hubert, & Wilson, 2009). Hence, we also aim to understand possible effects of a dysfunctional FA pathway on MIS.

## 2 MATERIALS AND METHODS

### 2.1 Ethical compliance

This study has been compliant with the ethical conduct of research and existing regulations.

### 2.2 Cell lines and DNA/RNA sample preparation

PD20 cell lines containing a defective *FANCD2* gene and retrovirally corrected *FANCD2* were obtained from the FA cell repository at the Oregon Health and Science University (https://www.ohsu.edu/research/fanconi-anemia/celllines.cfm/forum/index.cfm).

**Figure 1:**
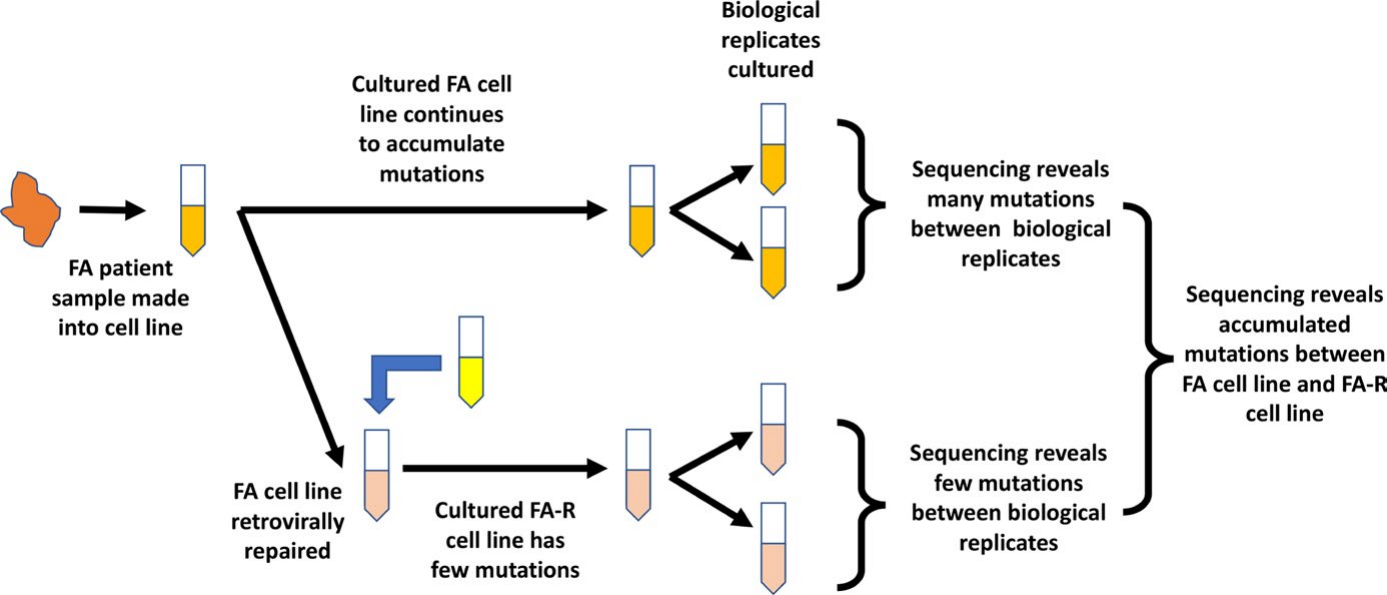
Two Fanconi anemia (FA)—PD20 cell lines were included in this experiment. The cell lines represented in yellow are FA cell lines with a dysfunctional *FANCD2* gene, while the cell lines represented in pink are FA cell lines that were corrected with a functional *FANCD2* gene using retroviral transduction. Variants with respect to the human genome reference found in common among all samples are indicative of variants accumulated prior to extraction from the patient and differences between this individual and the reference. Variants specifically distinguishing the FA and FA_RV sample groups are indicative of the divergence that likely occurred after retroviral correction in the FA_RV sample group. Variant differences within the sample groups (cell culture replicates) indicate the divergence of each of the cell line cultures, as different uncorrected errors accumulate in each sample

The cells were seeded onto six‐well plates at 1.5 × 10^4^ cells/well. After ~24 hr of incubation, the medium (5 ml of DMEM‐10% FBS) was changed. After 4 hr of incubation, the cells were trypsinized and collected for DNA (Qiagen AllPrep Kit) and RNA (Qiagen RNA prep kit) extraction. DNA and RNA from two PD20 samples and two PD20 RV corrected for FANCD2 gene were sent for exomic sequencing and RNA‐Seq analysis (Figure 1).

### 2.3 DNA variant detection

Paired‐end sequencing reads of the four Fanconi anemia DNA samples were obtained in the form of fastq files. The samples had a mean of 30 million reads with a standard deviation of 3 million reads. The samples were then checked for quality using the Trimmomatic tool (Bolger, Lohse, & Usadel, 2014) that utilizes a window‐based system to check for the sequencing quality of bases. The tool requires user input for three parameters: sliding window length, quality score threshold, and minimum length of sequencing read. The following default values were used: 10, 20, and 70. Only sequences that contained both mate pair reads were used for further processing. After quality trimming, each sample on average had 27 million reads with a standard deviation of 3 million. The “mem” function of the BWA package was used to map the sequencing reads to the GRCh38 reference genome (Li & Durbin, 2009). On average, 99% of reads in all the samples were mapped to the reference genome. The SAMtools package was used to convert the SAM file into a sorted and indexed BAM file (Li et al., 2009). The AddOrReplaceReadGroups function in the Picard package was used to add read groups to the BAM files. All four BAM files were pooled together to create INDEL realignment targets using the RealignerTargetCreator function in GATK package. The GATK IndelRealigner function was then used on individual samples to create INDEL realigned BAM files (McKenna et al., 2010). The GATK HaplotypeCaller with default parameter settings was used to create VCF files with INDEL and SNP calls, and the SelectVariants function was used to separate SNPs and INDELs. The latter were detected in the size range of 1–10 bp.

Python and shell scripts were written to calculate the number of commonly present SNPs and INDELs in the FA and FA_RV samples. The BEDTOOLS coverage function was used to calculate the percentage of the exome that is covered by the reads and the SAMtools depth function (samtools depth sample.bam|awk '{sum += $3} END {print "Average = ",sum/NR}') was used to calculate the coverage of the reads covering the exome (Quinlan & Hall, 2010).

The VCF files were fed to the SNPEff program to annotate the SNP and INDEL calls (Cingolani et al., 2012). The SNPEff annotation output was used to classify the detected variants according to the type and genomic region. The SNPEff program outputs the possible effects of a given variant and classifies the effect into four categories: high, low, moderate, and modifier.

Custom‐written Python scripts were used to perform the pairwise comparison of DNA variants that were predicted as high impact by SNPEff. A SNP is considered to be specific to a sample A, if the altered nucleotide is covered by at least three reads in sample A and not present even once in sample B. This base‐by‐base comparison was done only for nucleotide positions that were covered by at least 10 reads in both samples. The SAMtools mpileup function was utilized to check whether genomic positions that span a variant call in one sample are covered in the other sample. Control biological replicate exomes (SRR2776256 and SRR2776257), to demonstrate the validity of this SNP comparison procedure, were obtained elsewhere (Linderman et al., 2014).

### 2.4 RNA‐Seq data processing and analysis

Single‐end sequencing reads from RNA samples were obtained in the form of fastq files. On average, each sample contained 36 million reads with a standard deviation of 1 million. They were quality checked and trimmed using the Trimmomatic tool with the same parameters that were used for the DNA samples. After quality trimming, the samples retained on average 35 million reads (*SD* 1 million) (Bolger et al., 2014). The quality‐trimmed RNA sequence reads were then mapped to the GRCh38 reference genome using Tophat2 with a prebuilt transcriptome index (Trapnell, Pachter, & Salzberg, 2009). On average, 90% (*SD*: 3%) of the reads were uniquely mapped. The mapped BAM file was passed through the Cufflinks and Cuffmerge programs and was finally passed through the Cuffdiff program as four separate sample groups (Trapnell et al., 2010). The Cuffdiff program generates a pairwise comparison of the FPKM (Fragments Per Kilobase of transcript per Million mapped reads) values for each gene in all four samples, providing six pairwise comparisons. Any gene that was found to have a statistically significant (*p* value < 0.05) differential expression in comparisons across the FA and FA_RV sample groups, and no significant differential expression within the FA and FA_RV sample groups, was considered to be differentially expressed between the two sample groups.

The Benjamini–Hochberg false discovery rate for the differential expression of the *FANCD2* gene was calculated by separately computing the false discovery rate (FDR) for all six possible comparisons of the four samples. These *p*‐values were extracted from the gene_exp.diff file which is one of the output files that the Cuffdiff program generates. The FDR was computed as follows: Three values were utilized. (a) *p* value, (b) total number of tests, and (c) rank of that *p* value when sorted in ascending order. The formula for calculating FDR = *p* value * (total tests/*p* value rank). The genes.fpkm_tracking file was used to count the number of exons for each gene using its most expressed transcript.

### 2.5 Microsatellite Genotyping

Microsatellite (MST) genotyping of the DNA samples was performed using the Repeatseq program (Highnam et al., 2013). The program requires three user inputs: mapped sequencing reads in the form of a BAM file, a list of MST genomic coordinates, and the reference genome. Repeatseq outputs a variant call file listing all possible alleles detected for each microsatellite locus. Custom‐written Perl scripts were written to generate a list of MSTs that include primary, secondary, and minor allele information. We followed our established genotyping method to classify a microsatellite into homozygous and heterozygous genotypes (McIver, Fonville, Karunasena, & Garner, 2014; McIver, McCormick, Martin, Fondon, & Garner, 2013). A MST is considered to have a minor allele only if the minor allele was covered by at least three reads.

### 2.6 Generating the query list of microsatellites

A list of microsatellite loci in version 19 of the human reference genome was generated with a custom Perl script “searchTandemRepeats.pl” using default parameters. This script has been used in previous microsatellite studies and is available online at https://genotan.sourceforge.net/#_Toc324410847. The genomic coordinates for these microsatellite sequences were converted to their corresponding GRCh38 coordinates. The initial list generated with this script included 1,671,121 microsatellite loci. To mitigate the likelihood of improper read mapping between microsatellites, we removed all subsets of microsatellites possessing the same motif between identical 3′ and 5′ flanking regions. For example, the microsatellites “GCTGC(A)_34_CTTAG” and “GCTGC(A)_15_CTTAG” were preemptively removed from our initial list of microsatellites. The fact that there were many of these potentially ambiguous regions is not surprising considering microsatellites are often embedded in larger repetitive motifs, such as LINEs and SINEs. Our final filtered list included 611,515 microsatellite loci. Microsatellites that were covered by at least 10 reads were genotyped and classified into homozygous and heterozygous.

### 2.7 Gene ontology (GO) analysis

The DAVID online bioinformatics tool was used to perform the ontological gene enrichment analysis (Huang et al., 2007). The DAVID tool was applied to the 270 genes found to be differentially expressed between the FA and FA_RV sample groups. The Reactome pathway online tool was used to find the pathways in which the two sets of genomic variant‐associated genes are involved (Croft et al., 2014). The pathways that were found to have significant (FDR < 0.05) involvement were considered for analysis.

## 3 RESULTS AND DISCUSSION

### 3.1 Genomic variant analysis

The four DNA samples, two biological replicates for FA cell line and two biological replicates for the FA_RV cell line, were sequenced and mapped to the human genome version 38 reference to identify genomic variants, such as SNPs and INDELs, and assess their potential impact. Approximately 55% of the INDEL‐containing loci were found (callable) in all four samples while 65% of the SNP‐containing loci were commonly callable in all four samples (Table 1), thus implying their common origin, that is, that these variants existed in the clinical sample prior to creating the cell lines. About 70% of INDELs and 75% of SNPs were shared by samples within groups (Table 1). The remaining fraction of unshared variants indicate that the biological replicates continue to genetically diverge, that is, develop different variants that go uncorrected, providing a mechanism for enhanced heterogeneity.

**Table 1:** Variant calls for the two Fanconi anemia (FA) and two *FANCD2* RV‐corrected FA samples, with respect to the human reference genome

Variant type	Sample	Sample type	Variant count		
			No.	Repeated in replicates	Repeated in all samples
INDEL	GRL1398	FA	28,053	19,556	15,023
	GRL1399	FA	27,218		
	GRL1400	FA_RV	27,386	18,836	
	GRL1401	FA_RV	25,966		
SNP	GRL1398	FA	201,940	153,043	128,179
	GRL1399	FA	198,816		
	GRL1400	FA_RV	194,954	144,266	
	GRL1401	FA_RV	191,593		

Note

While a significantly large number of SNPs and INDELs were found to be repeated in all four samples, the difference in the number of variants within biological replicates shows evidence of heterogeneity in these replicate cell line cultures.

Genomic variants in both sample groups were annotated according to their various effects on genes and gene expression (Supporting information Table S1). Variants, such as start‐lost, stop‐gained, frameshift variant, which can immediately affect gene expression, were detected in both sample groups.

A significant increase in the fraction of INDELs in all four samples was observed, in comparison with the 1,000 Genome Project samples (1 kGP; Table 2) used as non‐FA controls. Chromosomal aberrations such as DNA lesions are signature variants of Fanconi anemia (Meyer et al., 2012). The increased fraction of INDEL variants observed in all four FA samples was consistent with dysfunctional correction mechanisms in FA. The fraction of the exome having been sequenced and the sequencing depth were approximately equal in the four FA samples and the 1 kGP samples. The SNP: INDEL ratios in the three different 1 kGP samples were consistently 9.2:0.8, while all FA samples had a higher fraction of INDELs (8.8:1.2).

**Table 2:** Distribution of SNPs and INDELs in the Fanconi anemia (FA), FA_RV, and 1 kGP samples

Sample	Sample Type	Total variants	SNP	INDEL	SNP–INDEL ratio	Exome % covered	Coverage
GRL1398	FA1	234,244	205,613	28,631	8.8:1.2	92	13
GRL1399	FA2	229,972	202,186	27,786	8.8:1.2	92	10
GRL1400	FA_RV_1	226,132	198,180	27,952	8.8:1.2	92	11
GRL1401	FA_RV_2	221,086	194,671	26,415	8.8:1.2	91	11
HG02003	1 KG	281,236	258,715	22,521	9.2:0.8	86	12
HG02008	1 KG	271,595	250,147	21,448	9.2:0.8	87	10
HG02009	1 KG	252,543	232,261	20,282	9.2:0.8	86	11
HG02010	1 KG	278,559	255,466	23,093	9.2:0.8	86	11

Note

While the ratio of SNPs versus INDELs in the FA and FA_RV samples is constant, an increase in the INDEL events is seen in the FA and FA_RV samples relative to the 1kGP samples, which was included as another type of control.

A set of 82 genes was associated with high‐impact genomic variants found only in the FA samples and a set of 618 genes was associated with high‐impact genomic variants found commonly in all four samples (FA and FA_RV sample groups), including 17 in 16 DNA repair‐related genes (Supporting information Table S2). To explore in detail the heterogeneity in these four samples, pairwise comparisons of SNP occurrences were made (Table 3). SNP‐containing loci were compared only if the corresponding genomic location in the corresponding replicate sample was sequenced at sufficient depth for the genotype to be called. Pairwise comparison (six pairs; 12 comparisons) of high‐impact SNPs within and across the two sample groups shows that FA_RV2 sample had the highest number of sample‐specific accumulated single nucleotide mutations. Pairwise comparison of high‐impact SNPs of FA_RV2 with FA1, FA2, and FA_RV1 were 40, 27, and 27, respectively (Supporting information Table S3). A similar pairwise comparison for high‐impact SNPs was performed on publicly available human biological replicate control exome samples and the two samples contained, on average, two specific mutations, which elucidates the validity of this comparison procedure.

**Table 3:** Pairwise comparisons of high‐impact SNPs in Fanconi anemia (FA) and FA_RV samples show the extent of SNP variability within biological replicates

	FA1	FA2	FA_RV1	FA_RV2
FA1	‐	4	20	30
FA2		‐	22	23
FA_RV1			‐	17
FA_RV2				‐

Note

Only SNPs called with respect to the reference genome that was sequenced in both samples in a sample pair were considered. Each of the six values in the matrix above represents the mean of two pairwise comparisons.

These accumulated mutations in FA_RV2 show that most of the SNP variation have accumulated prior to retroviral correction of the *FANCD2* gene. It should be noted that such high levels of variance in the genome are consistent with the fact that FA patients are highly predisposed to cancer (Nalepa & Clapp, 2018). Uncorrected interstrand cross‐links can directly affect DNA replication by phenomenally increasing DNA errors which can lead to cell death or uncontrolled cell growth (Osawa, Davies, & Hartley, 2011). Unlike SNPs, INDELs were more prevalent in FA than FA_RV samples, suggesting that INDELs may have continued to accumulate in the *FANCD2*‐uncorrected cell lines. *FANCD2* deficiency is related to replication fork restart defects (Thompson et al., 2017), and stalled replication forks have been known to result in INDELs (Sankar, Wastuwidyaningtyas, Dong, Lewis, & Wang, 2016). However, it should be noted that this analysis does not rule out effects of other genome repair checkpoints or even side effects of cell immortalization. A total of 17 high‐impact mutations in 16 genes from a variety of DNA repair pathways were indeed found in all four samples (Supporting information Table S2).

Microsatellite genotyping is used to understand the effect of a defective cross‐link repair mechanism on MST instability. It has been suggested that MST instability is not only caused by the mismatch repair mechanism defects, but could also be caused by defects in nucleotide excision repair and cross‐link repair mechanisms (Concannon & Lahue, 2014; Hubert et al., 2011; Lin et al., 2009). The fraction (5%, i.e., 256 MSTs) of heterozygous MSTs in the FA samples was found to be higher than the fraction (3.5%, i.e., 138 MSTs) in the 1 kGP samples, which is consistent with the increase in the fraction of MSTs with minor alleles (Table 4). The fraction of MSTs with minor alleles was 5% (200 MSTs) in the 1 kGP samples, while the fraction of MSTs with minor alleles was 8.5% (437 MSTs) in the FA samples (Table 4).

**Table 4:** Microsatellite genotyping of the four Fanconi anemia (FA) samples and healthy controls from the 1 kGP

Sample	Callable MST	Homozygous	Heterozygous	Minor alleles
FA1	5,395	95.4	4.6	7.6
FA2	5,011	94.9	5.1	7.7
FA_RV1	5,818	94.9	5.1	9.2
FA_RV2	4,519	95.0	5.0	9.3
HG02003	4,485	96.6	3.4	5.5
HG02008	3,834	97.1	2.9	4.5
HG02009	3,211	96.6	3.4	4.0
HG02010	4,158	95.6	4.4	6.1

Note

On average, 8.4% of the callable Microsatellite (MST) loci have minor alleles, while only 5% of the callable MSTs in 1 kGP samples have minor alleles, indicating a statistically higher rate of mutations in MSTs in the Fanconi anemia samples.

### 3.2 RNA sequencing to measure expression changes

Having examined the extent of genomic damage caused by a dysfunctional DNA repair gene, it was pertinent to examine the downstream effect of this DNA damage on genomewide gene expression patterns. A total of 270 genes were found to be differentially expressed in both biological replicates between the FA and the FA_RV sample groups (Table 5 and Supporting information Table S4). Significant fold changes in expression ranged from 1.1 to 11.5. Out of the 22 FA‐related genes, only *FANCD2* did have a significant gene expression change between FA and FA_RV sample groups (Table 6 and Supporting information Table S5). The PD20 cells are characterized by an amino acid change (S126G), mis‐splicing, and insertion of 13 bp from intron 5 into the *FANCD2* mRNA (Timmers et al., 2001). However, the relative difference in *FANCD2* expression between FA and FA_RV sample groups likely results from *FANCD2* overexpression in the corrected cells. Although thousands of genes had significant high‐impact variants in their coding and regulatory regions, only 270 genes were associated with significant expression shifts, indicating that although expression for a given gene was not changed the product structure was.

**Table 5:** Of the 270 differentially expressed genes, those with the highest fold change are illustrated here. Eight had a high gene expression fold change and were significantly divergent from the exponential distribution of the full set of genes, when comparing Fanconi anemia (FA) and FA_RV sample groups

#	Gene ID	Gene Symbol	Genomic position	GE fold change	High expression in FA_RV
1	XLOC_003069	−	chr1:239266341–239270392	11.5	+
2	XLOC_014162	COLEC12	chr18:318126–500729	10.5	−
3	XLOC_015674	ZNF626	chr19:20619938–20661596	10.0	−
4	XLOC_033910	FGF13	chrX:138631570–139222889	9.8	+
5	XLOC_011076	MT1E	chr16:56625653–56627112	9.5	−
6	XLOC_002609	GLUL	chr1:182381703–182392206	8.8	−
7	XLOC_018786	COL6A3	chr2:237324011–237422190	8.8	−
8	XLOC_002401	C1orf85	chr1:156292686–156295689	8.8	−

Note

A “+” indicates higher gene expression in FA_RV sample group, and a “−” indicates higher gene expression in FA sample group. See Supporting information Table S4 for the full list. See Supporting information Figure S1 for exponential fit graph.

**Table 6:** Pairwise comparisons of *FANCD2* gene expression in two Fanconi anemia (FA) samples and two *FANCD2* RV‐corrected FA samples

Sample 1	Sample 2	Sample 1 FPKM	Sample 2 FPKM	log2 (Fold change)	FDR
FA1	FA2	4.6	3.4	−0.4	5.320
FA1	FA_RV1	4.6	108.6	4.5	0.000
FA2	FA_RV1	3.4	108.6	5.0	0.000
FA1	FA_RV2	4.6	124.2	4.7	0.000
FA2	FA_RV2	3.4	124.2	5.1	0.000
FA_RV1	FA_RV2	108.6	124.2	0.1	4.870

Note

FPKM is the fragments per kilobase of exon per Million fragments mapped; FDR is false discovery rate.

Gene expression calculated by FPKM confirms that compared to FA samples, the expression of *FANCD2* in the FA_RV samples is significantly increased. This verifies the retroviral correction of the *FANCD2* gene in the FA_RV samples.

Structural and sequence variants have been known to influence the genes’ expression levels (Chiang et al., 2017; Li et al., 2017). Of the 82 genes that contain FA‐specific high‐impact DNA variants, one was also found to be a differentially expressed gene (*GUCY1B3*; OMIM #139397) (Supporting information Table S4). The C (Cytosine) at chromosome‐4: genomic position‐155789752 was found to be mutated into an A (Adenine), converting a TAC codon into a TAA (stop codon). This scenario, a single commonality between high‐impact DNA variants and genes with a significant gene expression difference, presents a critical opportunity to study the effect of a dysfunctional DNA repair‐caused mutation at the transcriptome level. The effect of the premature stop codon is a gene expression difference between the FA and FA_RV sample groups with a fold change of 5.07 (Supporting information Table S4). *GUCY1B3* was found to be upregulated in the FA_RV sample group, which is consistent with the expectation that the sample group (FA) with the high‐impact DNA variant will give rise to deterred gene expression.

The pairwise comparisons of the number of differentially expressed genes in all four samples showed that on average all four pairwise comparisons across sample groups had 375 differentially expressed genes (FA1 − FA_RV1 = 376; FA1 − FA_RV2 = 384; FA2 − FA_RV1 = 373; FA2 − FA_RV2 = 369), while the two comparisons within sample groups (FA1 − FA2 = 1, FA_RV1 − FA_RV2 = 1) had only one differentially expressed gene each. This confirms that although there was significant divergence between the genomes of the biological replicates, the effect at the transcriptome level was negligible, again suggesting that substantial changes to regulatory regions were rare or absent, perhaps because they were under significantly higher selection pressure. Also, as a control, and to put these numbers into context, three 1 kGP RNA‐Seq samples were downloaded and pairwise analyzed for differentially expressed genes. The number of differentially expressed genes in these three samples ranged from 105 to 236, significantly less than the number of genes whose expression changed in FA samples. It should be noted that the expression measurements in the 1 kGP samples were for different individuals under divergent conditions, while the FA samples were from the same individual under controlled culture conditions.

In addition to the raw expression level changes, there are indications that these genes are alternatively spliced. Pairwise comparisons of exon count in genes within and across the FA and FA_RV sample groups showed that on average 28.5% of expressed genes had varying exon counts across sample groups, while 23.5% of expressed genes had varying exon counts within sample groups. An increase of 5% of genes (795 genes) with varying exon counts between FA and FA_RV sample groups showed the effects of the dysfunctional FANCD2 gene on alternative splicing (Supporting information Table S6). These findings suggest that a dysfunctional DNA repair mechanism leads to DNA damage which in turn affects gene expression through truncated RNA transcripts rather than directly affecting the number of genes that are expressed.

### 3.3 GO analysis of genes with differential expression and/or genomic variants

To gain insights into the mechanistic role of genes that were affected by a DNA repair gene (*FANCD2*) dysfunction, two sets of genes were analyzed for their gene ontology term enrichment and their overrepresentation in pathways: (a) set of genes (270) were found to be differentially expressed (RNA‐Seq) between the FA and FA_RV sample groups; (b) set of genes (82) that were associated with high‐impact DNA variants that were specific to the FA sample group and were not found in the FA_RV sample group (Supporting information Table S7).

Pathway analysis of the FA‐specific gene set (82) shows involvement in a variety of functions, including apoptosis and transcriptional regulation, along with known immune‐related FA functions such as antigen presentation and immune‐related signaling (Supporting information Table S8). The common gene variant set (270), on the other hand, is predominantly involved in immune‐related pathway functions. This suggests that earlier mutations target the immune system while further mutations that are caused by the unrepaired FA pathway may lead to a wider variety of genomic effects initiating apoptosis.

Pathway analysis of the differentially expressed genes showed that signaling genes were specifically involved in immunological processes, including interferon cell signaling. One of the highly overrepresented pathways as indicated by the differentially expressed genes was the endosomal pathway (Supporting information Table S9). The cathepsin L gene (CTSL – OMIM #116880; FDR: 5.88E‐15) that is mainly involved in the lysosomal degradation of proteins was not only differentially expressed between the FA and FA_RV sample group, but it was upregulated in the FA samples and downregulated in the FA_RV samples. The upregulation of the CTSL gene that is directly involved in lysosomal activity is consistent with the increase in the production of broken transcripts (Supporting information Table S4), which can eventually lead to stalled elongation of protein molecules and ribophagy through lysosomes (Doma & Parker, 2006; Lafontaine, 2010).

Other overrepresented pathways were the PDCD‐1 (programmed cell death – FDR: 0.034) signaling pathway and ER‐phagosome pathway (FDR: 5.88E‐15). For example, one gene involved in the phagosome pathway was MYD88 (OMIM #602170; FDR: 6.35E‐12) which again was found to be more highly expressed in the FA sample group and not in the FA_RV sample group. We observed the upregulation of 12 collagen genes (COL4A2, COL5A2, COL1A2, COL6A2, COL6A1, COL8A1, COL11A1, COL4A1, COL3A1, COL16A1, COL5A3, COL6A3) in the FA sample group.

## 4 CONCLUSIONS

In this study, we quantified the variants from but one of the many (currently estimated at 22) genes that are members of the FA DNA repair complex and found many high‐impact variants that have accumulated as a consequence of uncorrected genomic mistakes. Although there were many sequence variants, relatively few genes with altered expression were found. Together, these sequence and expression changes occurred in genes which are typically associated with a spectrum of clinical disorders. The primary clinical disorders suffered by FA patients are consistent with the ones that ontological and pathway analyses would predict for the genes harboring the observed variants. For example, cell viability and the regulation of the extracellular matrix have been convincingly linked to cancer progression (Takamoto, Leppert, & Yu, 1998). The upregulation of a collection of collagen coding proteins we observed is consistent with upregulation of cell death signaling and phagosomal activity. Indeed, FA patients are predisposed to cancers, especially acute myeloid leukemia and squamous cell carcinomas (Mathew, 2006; Takamoto et al., 1998). These rapid genetic changes suggest that the clinical fate of any individual FA patient is likely determined by the unique spectrum of uncorrected stochastic variations accumulated during the lifetime of the patient. Overall, our results provide new insights into genome‐ and transcriptome‐wide effects of a dysfunctional DNA repair gene. Better understanding of the effects of this and other FA genes underlying downstream molecular events that ultimately lead to the disease‐causing phenotypical changes will have critical implications for future therapeutic strategies.

## CONFLICT OF INTEREST

The authors declare that there are no conflict of interests.

## AUTHOR CONTRIBUTIONS

KRV and LK performed the data analysis and interpreted the results. NCF cultured the cell lines and prepared DNA and RNA for analysis. PM, NCF, and HRG were responsible for experimental design. All authors contributed to manuscript writing and agree with it as submitted.

## DATA ACCESSIBILITY

Raw and processed data have been deposited at NCBI's SRA (#SRP162355).

## References

[mgg3502-bib-0001] Ameziane, N. , May, P. , Haitjema, A. , van de Vrugt, H. J. , van Rossum‐Fikkert, S. E. , Ristic, D. , … Dorsman, J. C. (2015). A novel Fanconi anaemia subtype associated with a dominant‐negative mutation in RAD51. Nature Communications, 6, 8829 10.1038/ncomms9829. PMC4703882 26681308

[mgg3502-bib-0002] Auerbach, A. D. (2009). Fanconi anemia and its diagnosis. Mutation Research, 668(1–2), 4–10. 10.1016/j.mrfmmm.2009.01.013. 19622403 PMC2742943

[mgg3502-bib-0003] Bogliolo, M. , & Surralles, J. (2015). Fanconi anemia: A model disease for studies on human genetics and advanced therapeutics. Current Opinion in Genetics & Development, 33, 32–40. 10.1016/j.gde.2015.07.002. 26254775

[mgg3502-bib-0004] Bolger, A. M. , Lohse, M. , & Usadel, B. (2014). Trimmomatic: A flexible trimmer for Illumina sequence data. Bioinformatics, 30(15), 2114–2120. 10.1093/bioinformatics/btu170. 24695404 PMC4103590

[mgg3502-bib-0005] Brulikova, L. , Hlavac, J. , & Hradil, P. (2012). DNA interstrand cross‐linking agents and their chemotherapeutic potential. Current Medicinal Chemistry, 19(3), 364–385. 22335513 10.2174/092986712803414295

[mgg3502-bib-0006] Che, R. , Zhang, J. , Nepal, M. , Han, B. , & Fei, P. (2018). Multifaceted Fanconi Anemia Signaling. Trends in Genetics, 34(3), 171–183. 10.1016/j.tig.2017.11.006. 29254745 PMC5858900

[mgg3502-bib-0007] Cheung, R. S. , & Taniguchi, T. (2017). Recent insights into the molecular basis of Fanconi anemia: Genes, modifiers, and drivers. International Journal of Hematology, 106(3), 335–344. 10.1007/s12185-017-2283-4. 28631178 PMC5904331

[mgg3502-bib-0008] Chiang, C. , Scott, A. J. , Davis, J. R. , Tsang, E. K. , Li, X. , Kim, Y. , … Hall, I. M. (2017). The impact of structural variation on human gene expression. Nature Genetics, 49(5), 692–699. 10.1038/ng.3834. 28369037 PMC5406250

[mgg3502-bib-0009] Cingolani, P. , Platts, A. , Wang le, L. , Coon, M. , Nguyen, T. , Wang, L. , … Ruden, D. M. (2012). A program for annotating and predicting the effects of single nucleotide polymorphisms, SnpEff: SNPs in the genome of Drosophila melanogaster strain w1118; iso‐2; iso‐3. Fly (Austin), 6(2), 80–92. 10.4161/fly.19695. 22728672 PMC3679285

[mgg3502-bib-0010] Concannon, C. , & Lahue, R. S. (2014). Nucleotide excision repair and the 26S proteasome function together to promote trinucleotide repeat expansions. DNA Repair (Amst), 13, 42–49. 10.1016/j.dnarep.2013.11.004. 24359926

[mgg3502-bib-0011] Croft, D. , Mundo, A. F. , Haw, R. , Milacic, M. , Weiser, J. , Wu, G. , … D'Eustachio, P. (2014). The Reactome pathway knowledgebase. Nucleic Acids Research, 42(D1), D472–D477. 10.1093/nar/gkt1102. 24243840 PMC3965010

[mgg3502-bib-0012] Deans, A. J. , & West, S. C. (2011). DNA interstrand crosslink repair and cancer. NatureReviews. Cancer, 11(7), 467–480. 10.1038/nrc3088. PMC3560328 21701511

[mgg3502-bib-0013] Doma, M. K. , & Parker, R. (2006). Endonucleolytic cleavage of eukaryotic mRNAs with stalls in translation elongation. Nature, 440(7083), 561–564. 10.1038/nature04530. 16554824 PMC1839849

[mgg3502-bib-0014] Donahue, S. L. , & Campbell, C. (2004). A Rad50‐dependent pathway of DNA repair is deficient in Fanconi anemia fibroblasts. Nucleic Acids Research, 32(10), 3248–3257. 10.1093/nar/gkh649. 15199173 PMC434453

[mgg3502-bib-0015] Highnam, G. , Franck, C. , Martin, A. , Stephens, C. , Puthige, A. , & Mittelman, D. (2013). Accurate human microsatellite genotypes from high‐throughput resequencing data using informed error profiles. Nucleic Acids Research, 41(1), e32 10.1093/nar/gks981. 23090981 PMC3592458

[mgg3502-bib-0016] Howlett, N. G. , Taniguchi, T. , Olson, S. , Cox, B. , Waisfisz, Q. , De Die‐Smulders, C. , & D'Andrea, A. D. (2002). Biallelic inactivation of BRCA2 in Fanconi anemia. Science, 297(5581), 606–609. 10.1126/science.1073834. 12065746

[mgg3502-bib-0017] Huang, D. W. , Sherman, B. T. , Tan, Q. , Kir, J. , Liu, D. , Bryant, D. , … Lempicki, R. A. (2007). DAVID bioinformatics resources: Expanded annotation database and novel algorithms to better extract biology from large gene lists. Nucleic Acids Research, 35, W169–W175. 10.1093/nar/gkm415. 17576678 PMC1933169

[mgg3502-bib-0018] Hubert, L. Jr , Lin, Y. , Dion, V. , & Wilson, J. H. (2011). Xpa deficiency reduces CAG trinucleotide repeat instability in neuronal tissues in a mouse model of SCA1. Human Molecular Genetics, 20(24), 4822–4830. 10.1093/hmg/ddr421. 21926083 PMC3221534

[mgg3502-bib-0019] Joenje, H. , & Patel, K. J. (2001). The emerging genetic and molecular basis of Fanconi anaemia. Nature Reviews Genetics, 2(6), 446–457. 10.1038/35076590. 11389461

[mgg3502-bib-0020] Kutler, D. I. , Singh, B. , Satagopan, J. , Batish, S. D. , Berwick, M. , Giampietro, P. F. , … Auerbach, A. D. (2003). A 20‐year perspective on the International Fanconi Anemia Registry (IFAR). Blood, 101(4), 1249–1256. 10.1182/blood-2002-07-2170 12393516

[mgg3502-bib-0021] Lafontaine, D. L. (2010). A 'garbage can' for ribosomes: How eukaryotes degrade their ribosomes. Trends in Biochemical Sciences, 35(5), 267–277. 10.1016/j.tibs.2009.12.006. 20097077

[mgg3502-bib-0022] Li, H. , & Durbin, R. (2009). Fast and accurate short read alignment with Burrows‐Wheeler transform. Bioinformatics, 25(14), 1754–1760. 10.1093/bioinformatics/btp324. 19451168 PMC2705234

[mgg3502-bib-0023] Li, H. , Handsaker, B. , Wysoker, A. , Fennell, T. , Ruan, J. , & Homer, N. … Genome Project Data Processing Subgroup (2009). The sequence alignment/map format and SAMtools. Bioinformatics, 25(16), 2078–2079. 10.1093/bioinformatics/btp352. 19505943 PMC2723002

[mgg3502-bib-0024] Li, X. , Kim, Y. , Tsang, E. K. , Davis, J. R. , Damani, F. N. , Chiang, C. , … Montgomery, S. B. (2017). The impact of rare variation on gene expression across tissues. Nature, 550(7675), 239–243. 10.1038/nature24267. 29022581 PMC5877409

[mgg3502-bib-0025] Lin, Y. , Hubert, L. Jr , & Wilson, J. H. (2009). Transcription destabilizes triplet repeats. Molecular Carcinogenesis, 48(4), 350–361. 10.1002/mc.20488. 18973172 PMC3671855

[mgg3502-bib-0026] Linderman, M. D. , Brandt, T. , Edelmann, L. , Jabado, O. , Kasai, Y. , Kornreich, R. , … Schadt, E. E. (2014). Analytical validation of whole exome and whole genome sequencing for clinical applications. BMC Medical Genomics, 7, 20 10.1186/1755-8794-7-20. 24758382 PMC4022392

[mgg3502-bib-0027] Mace‐Aime, G. , Couve, S. , Khassenov, B. , Rosselli, F. , & Saparbaev, M. K. (2010). The Fanconi anemia pathway promotes DNA glycosylase‐dependent excision of interstrand DNA crosslinks. Environmental and Molecular Mutagenesis, 51(6), 508–519. 10.1002/em.20548. 20120016

[mgg3502-bib-0028] Mathew, C. G. (2006). Fanconi anaemia genes and susceptibility to cancer. Oncogene, 25(43), 5875–5884. 10.1038/sj.onc.1209878. 16998502

[mgg3502-bib-0029] McIver, L. J. , Fonville, N. C. , Karunasena, E. , & Garner, H. R. (2014). Microsatellite genotyping reveals a signature in breast cancer exomes. Breast Cancer Research and Treatment, 145(3), 791–798. 10.1007/s10549-014-2908-8. 24838940 PMC4031393

[mgg3502-bib-0030] McIver, L. J. , McCormick, J. F. , Martin, A. , Fondon, J. W. 3rd , & Garner, H. R. (2013). Population‐scale analysis of human microsatellites reveals novel sources of exonic variation. Gene, 516(2), 328–334. 10.1016/j.gene.2012.12.068. 23274653 PMC3815531

[mgg3502-bib-0031] McKenna, A. , Hanna, M. , Banks, E. , Sivachenko, A. , Cibulskis, K. , Kernytsky, A. , … DePristo, M. A. (2010). The genome analysis toolkit: A mapreduce framework for analyzing next‐generation DNA sequencing data. Genome Research, 20(9), 1297–1303. 10.1101/gr.107524.110. 20644199 PMC2928508

[mgg3502-bib-0032] Meetei, A. R. , Levitus, M. , Xue, Y. , Medhurst, A. L. , Zwaan, M. , Ling, C. , … Joenje, H. (2004). X‐linked inheritance of Fanconi anemia complementation group B. Nature Genetics, 36(11), 1219–1224. 10.1038/ng1458. 15502827

[mgg3502-bib-0033] Meyer, S. , Neitzel, H. , & Tönnies, H. (2012). Chromosomal aberrations associated with clonal evolution and leukemic transformation in Fanconi Anemia: Clinical and biological implications. Anemia, 2012, 349837 10.1155/2012/349837. 22675616 PMC3366199

[mgg3502-bib-0034] Michl, J. , Zimmer, J. , & Tarsounas, M. (2016). Interplay between Fanconi anemia and homologous recombination pathways in genome integrity. The EMBO Journal, 35(9), 909–923. 10.15252/embj.201693860 27037238 PMC4865030

[mgg3502-bib-0035] Nalepa, G. , & Clapp, D. W. (2018). Fanconi anaemia and cancer: An intricate relationship. Nature Reviews Cancer, 18(3), 168–185. 10.1038/nrc.2017.116. 29376519

[mgg3502-bib-0036] Osawa, T. , Davies, D. , & Hartley, J. A. (2011). Mechanism of cell death resulting from DNA interstrand cross‐linking in mammalian cells. Cell Death & Disease, 2, e187 10.1038/cddis.2011.70. 21814285 PMC3181417

[mgg3502-bib-0037] Quinlan, A. R. , & Hall, I. M. (2010). BEDTools: A flexible suite of utilities for comparing genomic features. Bioinformatics, 26(6), 841–842. 10.1093/bioinformatics/btq033. 20110278 PMC2832824

[mgg3502-bib-0038] Radhakrishnan, A. , & Green, R. Connections underlying translation and mRNA stability. Journal of Molecular Biology, 428(18), 3558–3564. 10.1016/j.jmb.2016.05.025. 27261255

[mgg3502-bib-0039] Sankar, T. S. , Wastuwidyaningtyas, B. D. , Dong, Y. , Lewis, S. A. , & Wang, J. D. (2016). The nature of mutations induced by replication‐transcription collisions. Nature, 535(7610), 178–181. 10.1038/nature18316. 27362223 PMC4945378

[mgg3502-bib-0040] Silva, J. M. , Perez, D. S. , Pritchett, J. R. , Halling, M. L. , Tang, H. , & Smith, D. I. (2010). Identification of Long stress‐induced non‐coding transcripts that have altered expression in cancer. Genomics, 95(6), 355–362. 10.1016/j.ygeno.2010.02.009. 20214974

[mgg3502-bib-0041] Takamoto, N. , Leppert, P. C. , & Yu, S. Y. (1998). Cell death and proliferation and its relation to collagen degradation in uterine involution of rat. Connective Tissue Research, 37(3–4), 163–175. 10.3109/03008209809002436 9862218

[mgg3502-bib-0042] Taniguchi, T. , & D'Andrea, A. D. (2006). Molecular pathogenesis of Fanconi anemia: Recent progress. Blood, 107(11), 4223–4233. 10.1182/blood-2005-10-4240. 16493006

[mgg3502-bib-0043] Thompson, E. L. , Yeo, J. E. , Lee, E. A. , Kan, Y. , Raghunandan, M. , Wiek, C. , … Sobeck, A. (2017). FANCI and FANCD2 have common as well as independent functions during the cellular replication stress response. Nucleic Acids Research, 45(20), 11837–11857. 10.1093/nar/gkx847. 29059323 PMC5714191

[mgg3502-bib-0044] Timmers, C. , Taniguchi, T. , Hejna, J. , Reifsteck, C. , Lucas, L. , Bruun, D. , … Grompe, M. (2001). Positional cloning of a novel Fanconi anemia gene, FANCD2. Molecular Cell, 7(2), 241–248. 10.1016/S1097-2765(01)00172-1 11239453

[mgg3502-bib-0045] Trapnell, C. , Pachter, L. , & Salzberg, S. L. (2009). TopHat: Discovering splice junctions with RNA‐Seq. Bioinformatics, 25(9), 1105–1111. 10.1093/bioinformatics/btp120. 19289445 PMC2672628

[mgg3502-bib-0046] Trapnell, C. , Williams, B. A. , Pertea, G. , Mortazavi, A. , Kwan, G. , van Baren, M. J. , … Pachter, L. (2010). Transcript assembly and quantification by RNA‐Seq reveals unannotated transcripts and isoform switching during cell differentiation. Nature Biotechnology, 28(5), 511–515. 10.1038/nbt.1621. PMC3146043 20436464

