# Supplementary figures and images for: Dysfunctional DNA repair pathway via defective FANCD2 gene engenders multifarious exomic and transcriptomic effects in Fanconi anemia
Source: Mol Genet Genomic Med. 2018 Nov 18;6(6):1199–208. doi: 10.1002/mgg3.502 (PMC6305641; doi:10.1002/mgg3.502)

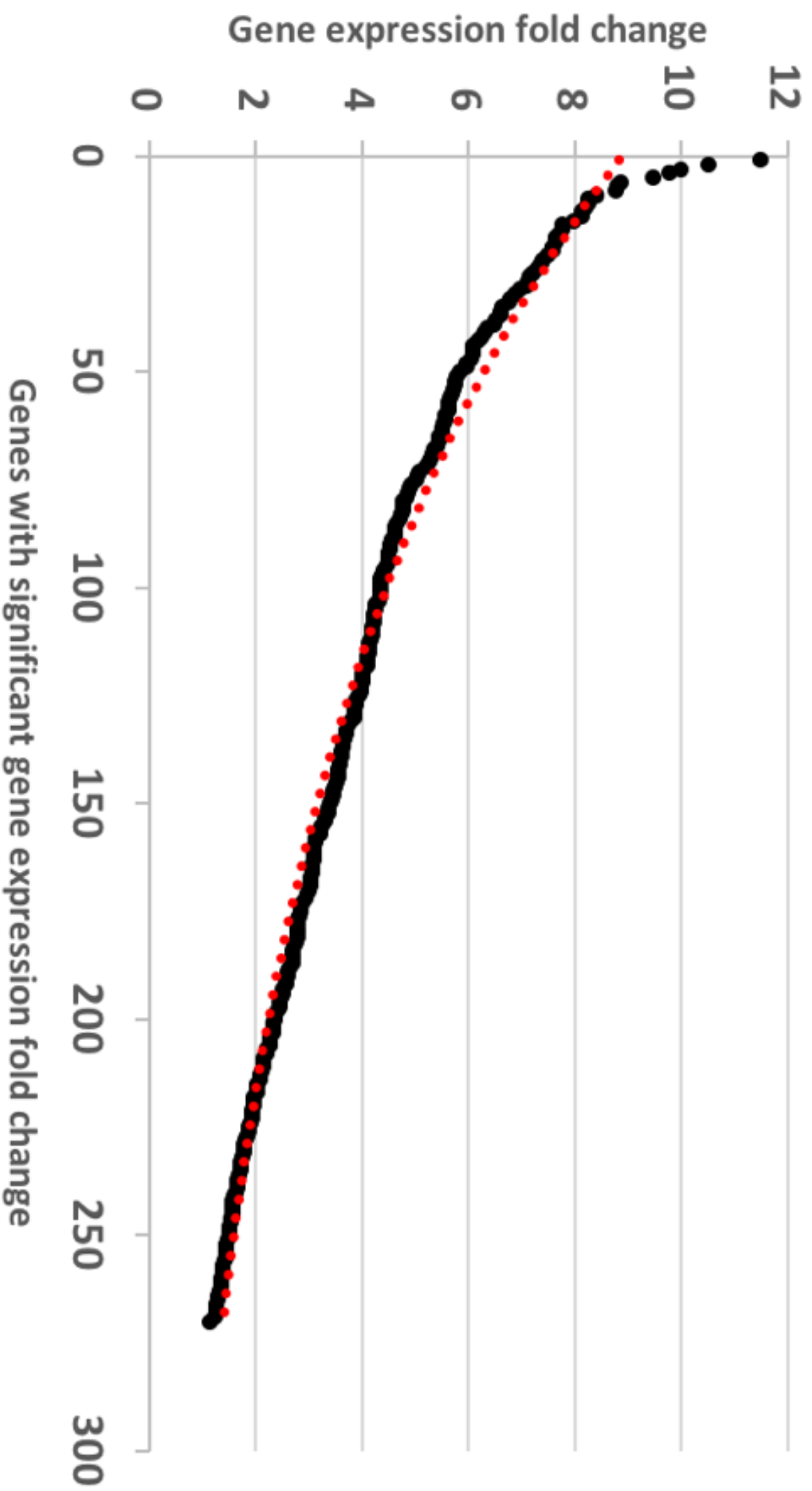

Supplement: Supplementary file 1 [file MGG3-6-1199-s001.pdf]
