# Supplementary material for: Dysfunctional DNA repair pathway via defective FANCD2 gene engenders multifarious exomic and transcriptomic effects in Fanconi anemia
Source: Mol Genet Genomic Med. 2018 Nov 18;6(6):1199–208. doi: 10.1002/mgg3.502 (PMC6305641; doi:10.1002/mgg3.502)
Supplement: Supplementary file 2 [file MGG3-6-1199-s002.docx]

**Table S1: Distribution of the DNA variants with respect to the human genome reference (build - Grch38) in all four Fanconi Anemia samples.** There were a significant number of variants found, many that are high impact. The classification of variants was done using the SNPEff program that annotates GATK VCF output. This also illustrates that many variants had already been acquired when the primary cells were taken from the patient to establish the defective and corrected cell lines.

| # | DNA variant event | FA1 | FA2 | FA_RV1 | FA_RV2 |
| --- | --- | --- | --- | --- | --- |
| 1 | intron_variant | 664039 | 650794 | 644703 | 631624 |
| 2 | downstream_gene_variant | 289232 | 283457 | 278135 | 276387 |
| 3 | upstream_gene_variant | 200121 | 194620 | 190464 | 188488 |
| 4 | 3_prime_UTR_variant | 59656 | 59324 | 57570 | 57931 |
| 5 | non_coding_transcript_exon_variant | 59060 | 58437 | 56577 | 56950 |
| 6 | synonymous_variant | 33460 | 33362 | 32162 | 32662 |
| 7 | missense_variant | 30275 | 30518 | 29686 | 31696 |
| 8 | intergenic_region | 30699 | 30131 | 29501 | 27426 |
| 9 | splice_region_variant | 14149 | 14279 | 13791 | 14389 |
| 10 | 5_prime_UTR_variant | 13877 | 13618 | 13058 | 13405 |
| 11 | sequence_feature | 5716 | 5569 | 5509 | 5455 |
| 12 | structural_interaction_variant | 2965 | 3044 | 2998 | 2987 |
| 13 | 5_prime_UTR_premature_start_codon_gain_variant | 1855 | 1815 | 1746 | 1831 |
| 14 | TF_binding_site_variant | 1175 | 1074 | 1120 | 1130 |
| 15 | frameshift_variant | 643 | 650 | 647 | 727 |
| 16 | stop_gained | 354 | 410 | 368 | 767 |
| 17 | splice_acceptor_variant | 391 | 424 | 343 | 428 |
| 18 | splice_donor_variant | 361 | 308 | 343 | 367 |
| 19 | disruptive_inframe_deletion | 317 | 307 | 311 | 312 |
| 20 | disruptive_inframe_insertion | 227 | 229 | 219 | 210 |
| 21 | conservative_inframe_insertion | 170 | 149 | 167 | 176 |
| 22 | protein_protein_contact | 154 | 154 | 154 | 153 |
| 23 | conservative_inframe_deletion | 72 | 101 | 89 | 66 |
| 24 | start_lost | 70 | 76 | 61 | 68 |
| 25 | stop_lost | 52 | 52 | 56 | 55 |
| 26 | stop_retained_variant | 46 | 47 | 39 | 41 |
| 27 | non_coding_transcript_variant | 37 | 27 | 30 | 23 |
| 28 | intragenic_variant | 24 | 20 | 19 | 19 |
| 29 | TFBS_ablation | 10 | 15 | 11 | 5 |
| 30 | initiator_codon_variant | 4 | 4 | 4 | 4 |
| 31 | bidirectional_gene_fusion | 2 | 2 | 3 | 3 |
| 32 | gene_fusion | 1 | 1 | 1 | 1 |

**Table S2: High impact SNPs within DNA-repair-related genes.**

| **Gene** | **CHR** | **Position** | **Ref Allele** | **Alt Allele** | **Mutation Type** | **FA1** | **FA2** | **FA_RV1** | **FA_RV2** |
| --- | --- | --- | --- | --- | --- | --- | --- | --- | --- |
| AHCY | chr7 | 129400284 | T | TC | splice_acceptor_variant, intron_variant | 0/1 | 0/1 | 1/1 | 1/1 |
| ALKBH3 | chr11 | 43919094 | G | T | structural_interaction_variant | 1/1 | 1/1 | 1/1 | 1/1 |
| EME1 | chr17 | 50378624 | A | T | structural_interaction_variant | 1/1 | 1/1 | 1/1 | 1/1 |
| ERCC6 | chr9 | 95876006 | A | G | start_lost | 0/1 | 0/1 | 0/1 | 0/1 |
| FANCA | chr16 | 89770464 | T | C | splice_acceptor_variant, intron_variant | 1/1 | 1/1 | 1/1 | 1/1 |
| KPNA2 | chr17 | 68043234 | A | G | structural_interaction_variant | 0/1 | 0/1 | 0/1 | 0/1 |
| LDHA | chr11 | 18402904 | C | T | structural_interaction_variant | 1/1 | 1/1 | 1/1 | 1/1 |
| LIG4 | chr13 | 108211243 | G | A | structural_interaction_variant | 0/1 | 0/1 | 1/1 | 1/1 |
| MUS81 | chr11 | 65864791 | G | T | structural_interaction_variant | 0/1 | 0/1 | 0/1 | 0/1 |
| MUS81 | chr11 | 65864502 | G | A | structural_interaction_variant | 0/1 | 0/1 | 0/1 | 0/1 |
| NUDT1 | chrX | 51496443 | ATCCTCGAGGCAGCC | A | frameshift_variant, start_lost | 1/1 | 1/1 | 1/1 | 1/1 |
| RAD23B | chr9 | 107331689 | CA | C, CAA | frameshift_variant | 1/2 | 1/2 | 1/2 | 1/2 |
| RPA1 | chr17 | 1879663 | C | T | structural_interaction_variant | 0/1 | 0/1 | 0/1 | 0/1 |
| RPL27 | chr17 | 50368313 | CCTGGTCAG | C | splice_acceptor_variant, splice_region_variant, intron_variant | 0/1 | 1/1 | 1/1 | 1/1 |
| SND1 | chr7 | 128081455 | T | C | structural_interaction_variant | 1/1 | 1/1 | 1/1 | 1/1 |
| TDG | chr12 | 103979950 | G | GA | frameshift_variant | 0/1 | 0/1 | 0/1 | 0/1 |
| UBE2N | chrX | 143884366 | G | T | stop_lost, splice_acceptor_variant, splice_donor_variant, intron_variant | 1/1 | 1/1 | 1/1 | 1/1 |

**Mutation type:** We can reference the SNPEff tool for the detailed description of the mutation types.

**Genotype:** The two-number combination represent the two alleles. 0 represents reference allele. 1 represents the alternative allele. 2 represents the 2^nd^ alternative allele.

The complete list of the 246 DNA repair-related genes inspected: ACLY, PARP1, AHCY, APEX1, ATM, ATR, BLM, BRCA1, BRCA2, CANX, CCNH, CDC2, CDK7, CDKN3, CETN2, CHEK1, ERCC8, CKS2, COL1A2, CRIP2, DDB1, DDB2, DNMT1, TRDMT1, DNMT3A, DNMT3B, DUT, DVL3, E2F5, ERCC1, ERCC2, ERCC3, ERCC4, ERCC5, ERCC6, EZH2, FANCA, FANCC, FANCD2, FANCE, FANCB, FANCF, FANCG, FAP, FEN1, XRCC6, MSH6, GTF2H1, GTF2H2, GTF2H3, GTF2H4, H2AFX, H2AFZ, HDAC1, HDAC2, HNRPA2B1, PRMT1, HSPD1, HSPE1, HUS1, IARS, IFNGR2, ILF2, KPNA2, LDHA, LIG1, LIG3, LIG4, MBD1, MCM3, MECP2, MGMT, MLH1, MLL, MMP9, MNAT1, MPG, MRE11A, MSH2, MSH3, MSH4, MSH5, NUDT1, MUTYH, NBN, NME1, NONO, NTHL1, OGG1, PAFAH1B3, PCNA, PLK1, PMS1, PMS2L3, PMS2, POLB, POLD1, POLE, POLG, POLH, PPP2R5C, PRKDC, PSMA1, PSMC4, PSME2, PTMA, RAD1, RAD9A, RAD17, RAD23A, RAD23B, RAD51, RAD51C, RAD51L1, RAD51L3, RAD52, RBM4, RECQL, REV3L, RFC4, RPA1, RPA2, RPA3, RPL13, RPL27, MRPS12, SDHC, HLTF, SMARCA4, SNRPE, SNRPF, SOX4, SSBP1, SSR1, SUV39H1, TARS, TDG, PRDX2, TGIF1, TOP2A, TP53, TP53BP1, HSP90B1, TSTA3, UBE2A, UBE2B, UBE2N, UBE2V2, UNG, WRN, XPA, XPC, XRCC1, XRCC2, XRCC3, XRCC4, XRCC5, ARMET, SHFM1, RAD54L, OGT, MBD4, MBD2, EXO1, COPB2, RECQL5, RECQL4, TRAF4, HDAC4, KIAA0101, EIF4A3, DCLRE1A, HDAC6, CHAF1A, PARP2, RAD50, G3BP1, MAD2L2, CARM1, PRDX4, CCT4, PAICS, POLQ, MTHFD2, KDELR2, DMC1, CHEK2, POLI, TREX2, MRPL3, RPL35, TREX1, PNKP, CBX3, NCBP2, CCT5, TPX2, NUP205, ZDHHC17, SMUG1, SPO11, RAD54B, MLH3, SND1, APEX2, UBE2S, POLL, POLM, RPA4, RRM2B, POLK, REV1, MBD3, APTX, FANCL, NEIL3, TMEM30A, TDP1, RAD18, XAB2, FANCM, MMS19L, DCLRE1C, DCLRE1B, NEIL1, SUV39H2, EHMT1, NHEJ1, HDAC11, MUS81, SETD7, HDAC10, BRIP1, DOT1L, HEL308, ALKBH2, C18orf37, EME1, RDM1, TUBB, ALKBH3, NEIL2, FLJ35220, POLN, SETD8, GTF2H5

**Table S3: Variability in high impact SNPs.** This table presents the calculation of pairwise SNP comparisons in and across the FA and RA_RV sample groups. The number of unique high impact SNPs that are found specific in each sample shows the heterogeneity of the Fanconi Anemia samples.

| **Sample pair** | **# high impact SNPs** | | **SNPs specific to sample** | | **Mean high impact** | **Mean specific SNPs** |
| --- | --- | --- | --- | --- | --- | --- |
|  | **S1** | **S2** | **S1** | **S2** |  |  |
| FA1 - FA2 | 810 | 808 | 2 | 6 | 809 | 4 |
| FA1 - FA_RV1 | 810 | 786 | 19 | 22 | 798 | 21 |
| FA1 - FA_RV2 | 810 | 960 | 20 | 40 | 885 | 30 |
| FA2 - FA_RV1 | 808 | 786 | 23 | 21 | 797 | 22 |
| FA2 - FA_RV2 | 808 | 960 | 20 | 27 | 884 | 24 |
| FA_RV1 - FA_RV2 | 786 | 960 | 8 | 27 | 873 | 18 |

S1 – First sample of the samples in the sample pair column. S2 – Second sample of the samples in the sample pair column.

**Table S4: A total of 270 genes were found to be statistically significantly differentially expressed between the FA and FA_RV sample groups.** The genes are sorted according to the gene expression fold change. This table furnishes the entire list of 270 genes that were differentially expressed. The very large number of genes, with very large expression changes illustrate the significant impact that the many variants have on the cell lines.

| **Gene Id** | **Gene symbol** | **Genomic position** | **GE fold change** | **High expression in FA_RV** |
| --- | --- | --- | --- | --- |
| XLOC_003069 | - | chr1:239266341-239270392 | 11.48 | + |
| XLOC_014162 | COLEC12 | chr18:318126-500729 | 10.50 | - |
| XLOC_015674 | ZNF626 | chr19:20619938-20661596 | 10.00 | - |
| XLOC_033910 | FGF13 | chrX:138631570-139222889 | 9.79 | + |
| XLOC_011076 | MT1E | chr16:56625653-56627112 | 9.46 | - |
| XLOC_002609 | GLUL | chr1:182381703-182392206 | 8.85 | - |
| XLOC_018786 | COL6A3 | chr2:237324011-237422190 | 8.80 | - |
| XLOC_002401 | C1orf85 | chr1:156292686-156295689 | 8.78 | - |
| XLOC_022046 | CDCP1 | chr3:45082273-45146422 | 8.39 | - |
| XLOC_019959 | MX1 | chr21:41420557-41459214 | 8.25 | - |
| XLOC_006994 | CLEC2B | chr12:9852368-9869859 | 8.25 | - |
| XLOC_017545 | GALNT5 | chr2:157257547-157314078 | 8.18 | - |
| XLOC_008950 | LOC440157 | chr14:19298728-19303582 | 8.14 | + |
| XLOC_022006 | SUSD5 | chr3:33149927-33219215 | 8.14 | - |
| XLOC_021235 | MYD88 | chr3:38138477-38143022 | 7.97 | - |
| XLOC_011492 | XYLT1 | chr16:17102323-17470881 | 7.77 | - |
| XLOC_008904 | LOC102723726,TNFAIP2 | chr14:103121351-103137439 | 7.76 | - |
| XLOC_008934 | CRIP1 | chr14:105481517-105488789 | 7.70 | - |
| XLOC_031419 | - | chr8:122168573-122171056 | 7.65 | + |
| XLOC_029150 | PEG10 | chr7:94656324-94669695 | 7.65 | - |
| XLOC_007989 | ZIC2 | chr13:99981771-99986765 | 7.57 | + |
| XLOC_008412 | FLJ39632 | chr14:19076243-19096796 | 7.57 | + |
| XLOC_015095 | RCN3 | chr19:49527617-49543634 | 7.48 | - |
| XLOC_015837 | ATP1A3 | chr19:41966475-41994276 | 7.40 | + |
| XLOC_032446 | SLC35D2 | chr9:96313436-96383710 | 7.35 | - |
| XLOC_030357 | C8orf48 | chr8:13566842-13568288 | 7.31 | - |
| XLOC_017040 | EMILIN1 | chr2:27078566-27086403 | 7.22 | - |
| XLOC_030680 | - | chr8:122139981-122168546 | 7.16 | + |
| XLOC_017096 | QPCT | chr2:37344609-37373322 | 7.13 | - |
| XLOC_024738 | ZNF354C | chr5:179060406-179083771 | 7.09 | - |
| XLOC_003948 | PCBD1 | chr10:70882279-70888784 | 6.97 | - |
| XLOC_008753 | IFI27 | chr14:94110732-94116699 | 6.87 | - |
| XLOC_002724 | C1orf116 | chr1:207018520-207032761 | 6.78 | - |
| XLOC_031978 | PHYHD1 | chr9:128920894-128942041 | 6.76 | - |
| XLOC_010362 | CYP1A1 | chr15:74719541-74725610 | 6.63 | + |
| XLOC_006805 | HSPB8 | chr12:119178789-119194746 | 6.59 | - |
| XLOC_011239 | PLCG2 | chr16:81779293-81958294 | 6.59 | - |
| XLOC_026014 | CUL9 | chr6:43182174-43224587 | 6.50 | - |
| XLOC_027033 | LAMA4 | chr6:112107930-112306683 | 6.47 | - |
| XLOC_000273 | THEMIS2 | chr1:27872542-27886685 | 6.37 | - |
| XLOC_015133 | CD33 | chr19:51225078-51240019 | 6.29 | + |
| XLOC_002809 | ITPKB | chr1:226631689-226739327 | 6.25 | - |
| XLOC_032358 | - | chr9:78848554-78863649 | 6.17 | + |
| XLOC_012078 | ZFP3 | chr17:5078458-5096374 | 6.09 | - |
| XLOC_023003 | LIMCH1 | chr4:41359606-41700044 | 6.08 | + |
| XLOC_012093 | XAF1 | chr17:6755836-6775647 | 6.07 | - |
| XLOC_022879 | STK32B | chr4:5051545-5501001 | 6.06 | - |
| XLOC_000296 | SERINC2 | chr1:31409564-31434680 | 5.97 | - |
| XLOC_028763 | FAM20C | chr7:192958-260774 | 5.96 | - |
| XLOC_007833 | FREM2 | chr13:38687035-38887131 | 5.85 | + |
| XLOC_020937 | - | chr22:15854177-15855201 | 5.77 | + |
| XLOC_009291 | FBLN5 | chr14:91869409-91947702 | 5.75 | - |
| XLOC_014387 | - | chr18:14858969-14863974 | 5.75 | + |
| XLOC_009235 | MLH3 | chr14:75013763-75051532 | 5.71 | - |
| XLOC_006696 | - | chr12:98289603-98305388 | 5.69 | + |
| XLOC_034013 | CLIC2 | chrX:155276206-155334681 | 5.66 | - |
| XLOC_005225 | H19,MIR675 | chr11:1995175-1997835 | 5.62 | + |
| XLOC_007091 | TMTC1 | chr12:29500812-29784759 | 5.62 | + |
| XLOC_013665 | - | chr17:41110001-41112904 | 5.62 | + |
| XLOC_021327 | KLHDC8B | chr3:49171565-49176486 | 5.56 | - |
| XLOC_004657 | SERPING1 | chr11:57597553-57614853 | 5.55 | - |
| XLOC_022019 | SCN5A | chr3:38548061-38649675 | 5.54 | + |
| XLOC_007107 | KIF21A | chr12:39293227-39443390 | 5.51 | + |
| XLOC_030536 | TRIM55 | chr8:66127042-66175485 | 5.50 | - |
| XLOC_005657 | CD248 | chr11:66314486-66317044 | 5.44 | - |
| XLOC_010530 | - | chr15:30488358-30490284 | 5.43 | - |
| XLOC_017645 | COL3A1 | chr2:188974372-189012746 | 5.42 | - |
| XLOC_012724 | C1QTNF1 | chr17:79019208-79049788 | 5.34 | - |
| XLOC_011438 | PPL | chr16:4882506-4937135 | 5.33 | - |
| XLOC_006569 | DTX3 | chr12:57604326-57609804 | 5.28 | - |
| XLOC_012992 | USP32P2 | chr17:18511261-18531380 | 5.25 | - |
| XLOC_019537 | TSPY26P | chr20:32186497-32190526 | 5.21 | - |
| XLOC_023332 | GUCY1B3 | chr4:155758973-155807631 | 5.07 | + |
| XLOC_005429 | PAMR1 | chr11:35431826-35530300 | 5.05 | - |
| XLOC_012186 | TRPV2 | chr17:16415541-16437003 | 5.00 | - |
| XLOC_019717 | LAMA5 | chr20:62309059-62367312 | 4.93 | - |
| XLOC_023129 | ANXA3 | chr4:78551587-78610451 | 4.89 | - |
| XLOC_021138 | FANCD2 | chr3:10026383-10108291 | 4.87 | + |
| XLOC_015509 | COL5A3 | chr19:9959560-10010471 | 4.84 | - |
| XLOC_029774 | NSUN5 | chr7:73302515-73308867 | 4.77 | - |
| XLOC_000113 | TNFRSF1B | chr1:12166942-12209220 | 4.76 | - |
| XLOC_011062 | MMP2 | chr16:55479168-55506674 | 4.76 | - |
| XLOC_025963 | MAPK13 | chr6:36130483-36144524 | 4.74 | - |
| XLOC_025635 | DSP | chr6:7540450-7586713 | 4.70 | - |
| XLOC_017001 | KCNS3 | chr2:17877846-17932985 | 4.65 | + |
| XLOC_004032 | PPP1R3C | chr10:91628439-91633101 | 4.63 | - |
| XLOC_030912 | NEFL | chr8:24950954-24956869 | 4.62 | - |
| XLOC_024221 | CCDC152 | chr5:42756805-42811922 | 4.61 | - |
| XLOC_004154 | ABLIM1 | chr10:114431109-114779903 | 4.56 | - |
| XLOC_022647 | BDH1 | chr3:197509782-197573323 | 4.53 | - |
| XLOC_015498 | ZNF560 | chr19:9466354-9498603 | 4.52 | - |
| XLOC_000682 | GSTM1 | chr1:109687795-109693745 | 4.49 | - |
| XLOC_002107 | GSTM3 | chr1:109733931-109741038 | 4.49 | - |
| XLOC_007460 | TMEM119 | chr12:108589845-108598118 | 4.48 | - |
| XLOC_019438 | FERMT1 | chr20:6074844-6123544 | 4.47 | - |
| XLOC_005789 | ME3 | chr11:86441022-86672636 | 4.41 | - |
| XLOC_032395 | SEMA4D | chr9:89360790-89498014 | 4.36 | - |
| XLOC_017690 | CDK15 | chr2:201790453-201895550 | 4.35 | - |
| XLOC_023402 | SLC25A4 | chr4:185143262-185150384 | 4.35 | - |
| XLOC_014991 | APOE | chr19:44905781-44909393 | 4.34 | - |
| XLOC_001078 | PRRX1 | chr1:170662727-170739400 | 4.34 | - |
| XLOC_033198 | FAM133A | chrX:93674012-93712274 | 4.33 | + |
| XLOC_020061 | TMPRSS15 | chr21:18268866-18477284 | 4.30 | + |
| XLOC_003939 | AIFM2 | chr10:70052600-70132934 | 4.26 | - |
| XLOC_009515 | SNRPN,SNURF | chr15:24823646-24978582 | 4.25 | - |
| XLOC_001304 | MARK1 | chr1:220528182-220664457 | 4.23 | + |
| XLOC_007768 | TNFRSF19 | chr13:23570369-23676105 | 4.22 | - |
| XLOC_019508 | NINL | chr20:25452696-25585531 | 4.21 | - |
| XLOC_008016 | TEX29 | chr13:111320667-111344247 | 4.20 | - |
| XLOC_029826 | SEMA3A | chr7:83957817-84492768 | 4.20 | - |
| XLOC_018505 | DPP4 | chr2:161992240-162074542 | 4.18 | - |
| XLOC_008768 | BDKRB2 | chr14:96204797-96244329 | 4.15 | - |
| XLOC_017714 | ADAM23 | chr2:206443543-206621130 | 4.14 | + |
| XLOC_020507 | APOBEC3G | chr22:39077004-39087743 | 4.14 | - |
| XLOC_025666 | GMPR | chr6:16238579-16295549 | 4.13 | - |
| XLOC_013997 | ANKRD30B | chr18:14748239-14854702 | 4.11 | + |
| XLOC_001631 | MFAP2 | chr1:16974501-16981586 | 4.09 | - |
| XLOC_014195 | PIEZO2 | chr18:10670189-11149534 | 4.09 | - |
| XLOC_019417 | ADAM33 | chr20:3667972-3682131 | 4.03 | - |
| XLOC_009672 | GCHFR | chr15:40764086-40767713 | 4.00 | - |
| XLOC_002292 | CTSK | chr1:150796207-150808441 | 4.00 | - |
| XLOC_011431 | CDIP1 | chr16:4510674-4538815 | 4.00 | - |
| XLOC_019053 | SPTLC3 | chr20:13008953-13169001 | 3.99 | - |
| XLOC_020436 | TCN2 | chr22:30607082-30627060 | 3.97 | - |
| XLOC_021152 | PPARG | chr3:12287849-12434356 | 3.95 | - |
| XLOC_009076 | FOXA1 | chr14:37589551-37595120 | 3.89 | + |
| XLOC_019744 | STMN3 | chr20:63639704-63654977 | 3.88 | - |
| XLOC_001759 | COL16A1 | chr1:31652246-31704242 | 3.86 | - |
| XLOC_002087 | COL11A1 | chr1:102876466-103108496 | 3.86 | - |
| XLOC_032002 | AIF1L | chr9:131096475-131123152 | 3.84 | + |
| XLOC_011603 | STX1B | chr16:30989255-31010508 | 3.79 | - |
| XLOC_018582 | FRZB | chr2:182833274-182866770 | 3.74 | - |
| XLOC_024507 | TGFBI | chr5:136028894-136063818 | 3.70 | - |
| XLOC_024998 | F2RL2 | chr5:76403254-76708132 | 3.70 | - |
| XLOC_007942 | SLAIN1 | chr13:77697736-77764242 | 3.66 | + |
| XLOC_029710 | GRB10 | chr7:50590062-50793462 | 3.65 | - |
| XLOC_032192 | ELAVL2 | chr9:23690098-23826344 | 3.64 | + |
| XLOC_016959 | RSAD2 | chr2:6877664-6898232 | 3.61 | - |
| XLOC_017085 | LTBP1 | chr2:32947153-33399509 | 3.60 | - |
| XLOC_032494 | ABCA1 | chr9:104781001-104928246 | 3.57 | - |
| XLOC_000275 | XKR8 | chr1:27959992-27968093 | 3.57 | - |
| XLOC_020790 | FOXRED2 | chr22:36487185-36507101 | 3.56 | + |
| XLOC_021231 | CTDSPL | chr3:37862152-37984469 | 3.54 | - |
| XLOC_013620 | SECTM1 | chr17:82321023-82334045 | 3.54 | - |
| XLOC_007808 | MEDAG | chr13:30882561-30932608 | 3.52 | - |
| XLOC_020014 | COL6A1 | chr21:45981748-46005049 | 3.50 | - |
| XLOC_019958 | MX2 | chr21:41362022-41408943 | 3.47 | - |
| XLOC_007542 | OASL | chr12:121020291-121039242 | 3.46 | - |
| XLOC_007614 | CHFR | chr12:132840351-132887618 | 3.42 | - |
| XLOC_002410 | BCAN | chr1:156640499-156661441 | 3.39 | - |
| XLOC_012316 | CCL2 | chr17:34255276-34257203 | 3.37 | - |
| XLOC_028862 | GPNMB | chr7:23246685-23275110 | 3.35 | - |
| XLOC_031933 | OLFML2A | chr9:124777137-124814891 | 3.30 | - |
| XLOC_022011 | TRANK1 | chr3:36826816-36945057 | 3.29 | - |
| XLOC_031238 | MTSS1 | chr8:124550769-124728507 | 3.25 | + |
| XLOC_006782 | OAS1 | chr12:112906933-112919907 | 3.22 | - |
| XLOC_002412 | CRABP2 | chr1:156699605-156713174 | 3.22 | - |
| XLOC_014114 | SERPINB2 | chr18:63887704-63903890 | 3.13 | - |
| XLOC_024819 | CMBL | chr5:10277594-10308056 | 3.13 | - |
| XLOC_020015 | COL6A2 | chr21:46098118-46132849 | 3.10 | - |
| XLOC_003369 | PLAU | chr10:73909968-73922777 | 3.10 | - |
| XLOC_008041 | LINC00452 | chr13:113883636-113926238 | 3.10 | - |
| XLOC_018250 | CAPG | chr2:85394747-85414074 | 3.08 | - |
| XLOC_032636 | CRAT | chr9:129094793-129110791 | 3.07 | - |
| XLOC_026169 | POU3F2 | chr6:98834703-98838790 | 3.06 | + |
| XLOC_022447 | HLTF | chr3:149029382-149102823 | 3.05 | - |
| XLOC_018202 | PAIP2B | chr2:71182663-71227103 | 3.04 | + |
| XLOC_021488 | COL8A1 | chr3:99638364-99799220 | 3.02 | - |
| XLOC_022698 | - | chr3:75483604-75489296 | 3.02 | + |
| XLOC_002345 | S100A4 | chr1:153543495-153545806 | 2.99 | - |
| XLOC_006784 | OAS2 | chr12:112978346-113011723 | 2.96 | - |
| XLOC_033765 | TMSB15A | chrX:102513681-102516771 | 2.94 | + |
| XLOC_018606 | SDPR | chr2:191834304-191847280 | 2.88 | + |
| XLOC_004976 | FAT3 | chr11:92224640-92896533 | 2.85 | + |
| XLOC_026946 | ELOVL4 | chr6:79914811-79947598 | 2.85 | - |
| XLOC_027008 | CD24 | chr6:106969830-106975454 | 2.83 | + |
| XLOC_015120 | EMC10 | chr19:50466787-50505802 | 2.80 | - |
| XLOC_010197 | FBN1 | chr15:48408305-48645788 | 2.80 | - |
| XLOC_000565 | IFI44 | chr1:78649791-78664078 | 2.80 | - |
| XLOC_031709 | PGM5 | chr9:68355188-68531061 | 2.80 | - |
| XLOC_015457 | C3 | chr19:6677834-6720651 | 2.77 | - |
| XLOC_004350 | EPS8L2 | chr11:706116-727727 | 2.74 | - |
| XLOC_003177 | KIAA1217 | chr10:23694745-24557525 | 2.71 | - |
| XLOC_018507 | FAP | chr2:162114440-162243535 | 2.70 | - |
| XLOC_000564 | IFI44L | chr1:78620381-78646255 | 2.70 | - |
| XLOC_004340 | IFITM1 | chr11:313990-315272 | 2.68 | - |
| XLOC_008248 | KCTD12 | chr13:76880168-76886405 | 2.68 | + |
| XLOC_026181 | AIM1 | chr6:106360807-106570460 | 2.64 | - |
| XLOC_002167 | TBX15 | chr1:118882758-118989556 | 2.61 | - |
| XLOC_004418 | TRIM22 | chr11:5689586-5710863 | 2.59 | - |
| XLOC_018593 | COL5A2 | chr2:189031914-189179879 | 2.58 | - |
| XLOC_000213 | EPHB2 | chr1:22710769-22915330 | 2.56 | - |
| XLOC_020308 | USP18 | chr22:18149953-18177397 | 2.52 | - |
| XLOC_029590 | IGF2BP3 | chr7:23310208-23470491 | 2.51 | - |
| XLOC_001732 | IFI6 | chr1:27666060-27672213 | 2.49 | - |
| XLOC_020801 | RAC2 | chr22:37225260-37244299 | 2.46 | - |
| XLOC_013434 | CYB561 | chr17:63432303-63446363 | 2.45 | - |
| XLOC_032599 | ANGPTL2 | chr9:126914773-127223166 | 2.43 | - |
| XLOC_031215 | ENPP2 | chr8:119557076-119673404 | 2.37 | - |
| XLOC_006963 | C1R | chr12:7080208-7092570 | 2.36 | - |
| XLOC_008336 | COL4A1 | chr13:110148962-110307149 | 2.34 | - |
| XLOC_017455 | INHBB | chr2:120346142-120351807 | 2.34 | + |
| XLOC_031995 | ASS1 | chr9:130444706-130501274 | 2.33 | - |
| XLOC_023921 | DDX60 | chr4:168216290-168318807 | 2.28 | - |
| XLOC_004926 | DGAT2 | chr11:75768732-75801536 | 2.27 | + |
| XLOC_004725 | FADS2 | chr11:61799624-61867354 | 2.27 | - |
| XLOC_005840 | MMP1 | chr11:102783675-102843611 | 2.20 | - |
| XLOC_012791 | NXN | chr17:799312-979775 | 2.19 | - |
| XLOC_023355 | CPE | chr4:165378944-165498330 | 2.15 | - |
| XLOC_017529 | KIF5C | chr2:148875222-149026759 | 2.14 | + |
| XLOC_019324 | CDH4 | chr20:61252425-61940617 | 2.14 | + |
| XLOC_009361 | AHNAK2 | chr14:104924849-104978357 | 2.12 | - |
| XLOC_006972 | SLC2A3 | chr12:7919227-7936296 | 2.07 | - |
| XLOC_000012 | ISG15 | chr1:1013466-1014540 | 2.07 | - |
| XLOC_026239 | GJA1 | chr6:121435598-121449727 | 2.03 | - |
| XLOC_022561 | CAMK2N2 | chr3:184249656-184293031 | 2.02 | + |
| XLOC_023165 | HERC6 | chr4:88378685-88443097 | 2.02 | - |
| XLOC_014654 | CD97 | chr19:14380590-14408725 | 1.96 | - |
| XLOC_026329 | SASH1 | chr6:148212113-148552049 | 1.96 | - |
| XLOC_030733 | LY6K | chr8:142700110-142726973 | 1.95 | + |
| XLOC_006783 | OAS3 | chr12:112938443-112973251 | 1.94 | - |
| XLOC_031767 | CTSL | chr9:87726058-87731469 | 1.93 | - |
| XLOC_027083 | MOXD1 | chr6:132296054-132401525 | 1.93 | - |
| XLOC_026400 | MLLT4 | chr6:167826916-167972023 | 1.91 | - |
| XLOC_002032 | GBP1 | chr1:89052303-89065360 | 1.88 | - |
| XLOC_005937 | MCAM | chr11:119308523-119318377 | 1.86 | + |
| XLOC_026130 | TPBG | chr6:82363205-82367422 | 1.85 | - |
| XLOC_018508 | IFIH1 | chr2:162267078-162318708 | 1.81 | - |
| XLOC_002314 | S100A10 | chr1:151982909-151994238 | 1.79 | - |
| XLOC_023670 | ANTXR2 | chr4:79901217-80073472 | 1.78 | - |
| XLOC_029195 | PCOLCE | chr7:100586332-100608175 | 1.77 | - |
| XLOC_026113 | CD109 | chr6:73694235-73828317 | 1.76 | - |
| XLOC_008010 | COL4A2 | chr13:110307283-110513027 | 1.72 | - |
| XLOC_003446 | IFIT1 | chr10:89392545-89406487 | 1.72 | - |
| XLOC_005802 | CHORDC1 | chr11:90200428-90223364 | 1.71 | + |
| XLOC_000513 | PGM1 | chr1:63593275-63660245 | 1.69 | - |
| XLOC_030901 | TNFRSF10D | chr8:23135587-23164030 | 1.66 | + |
| XLOC_002230 | TXNIP | chr1:145992434-145996631 | 1.66 | - |
| XLOC_008358 | GAS6 | chr13:113815609-113864073 | 1.65 | - |
| XLOC_005644 | RNASEH2C | chr11:65711995-65720938 | 1.62 | + |
| XLOC_013563 | LGALS3BP | chr17:78971252-78979979 | 1.60 | - |
| XLOC_029851 | SAMD9 | chr7:93099512-93118023 | 1.57 | - |
| XLOC_008580 | LGALS3 | chr14:55129216-55145430 | 1.57 | - |
| XLOC_005191 | IFITM3 | chr11:319672-320914 | 1.56 | - |
| XLOC_012598 | MRC2 | chr17:62627400-62693601 | 1.55 | - |
| XLOC_005821 | MRE11A | chr11:94417299-94493874 | 1.52 | + |
| XLOC_001666 | HSPG2 | chr1:21812264-21937257 | 1.52 | - |
| XLOC_006321 | EMP1 | chr12:13196667-13216774 | 1.51 | - |
| XLOC_003133 | OPTN | chr10:13100074-13138291 | 1.50 | - |
| XLOC_030737 | LY6E | chr8:143018484-143022410 | 1.50 | - |
| XLOC_007246 | ITGA5 | chr12:54395260-54419266 | 1.48 | - |
| XLOC_025847 | HLA-A | chr6:29887759-29945884 | 1.45 | - |
| XLOC_003443 | IFIT2 | chr10:89301948-89309276 | 1.44 | - |
| XLOC_032210 | DDX58 | chr9:32455301-32526324 | 1.44 | - |
| XLOC_004997 | CEP57 | chr11:95790460-95832693 | 1.44 | + |
| XLOC_026696 | HLA-B | chr6:31353871-31357212 | 1.42 | - |
| XLOC_030925 | CLU | chr8:27596916-27615031 | 1.39 | - |
| XLOC_019496 | CST3 | chr20:23627896-23638048 | 1.38 | - |
| XLOC_025239 | DPYSL3 | chr5:147390807-147510056 | 1.37 | - |
| XLOC_002343 | S100A6 | chr1:153534596-153536241 | 1.36 | - |
| XLOC_029148 | COL1A2 | chr7:94394560-94431232 | 1.35 | - |
| XLOC_013255 | VAT1 | chr17:43014566-43022442 | 1.35 | - |
| XLOC_021728 | MME | chr3:155079646-155183729 | 1.32 | - |
| XLOC_005507 | UBE2L6 | chr11:57551654-57568330 | 1.30 | - |
| XLOC_005851 | CASP4 | chr11:104942866-104968598 | 1.28 | + |
| XLOC_005005 | YAP1 | chr11:102110419-102233423 | 1.27 | + |
| XLOC_009729 | EID1 | chr15:48823678-48963507 | 1.26 | - |
| XLOC_019547 | E2F1 | chr20:33675485-33686404 | 1.25 | + |
| XLOC_002315 | S100A11 | chr1:152032505-152037035 | 1.22 | - |
| XLOC_004759 | STIP1 | chr11:64185271-64204548 | 1.13 | + |

**Table S5: The log2(fold change) in gene expression for all the genes (22) that are known to be related to Fanconi Anemia for all six pairwise comparisons of two FA cell lines and FANCD2 RV corrected FA cell lines.** Only FANCD2 gene shows significant fold change in gene expression.

| **Gene** | **Gene expression fold change** | | | | | |
| --- | --- | --- | --- | --- | --- | --- |
|  | **FA1/** | **FA1/** | **FA2/** | **FA1/** | **FA2/** | **FA_RV1/** |
|  | **FA2** | **FA_RV1** | **FA_RV1** | **FA_RV2** | **FA_RV2** | **FA_RV2** |
| BRCA1 | 0.11 | 0.1 | -0.02 | 0.03 | -0.08 | -0.06 |
| BRCA2 | 0.12 | -0.18 | -0.3 | -0.06 | -0.18 | 0.12 |
| FANCD2 | -0.45 | 4.54 | 5 | 4.73 | 5.19 | 0.19 |
| PALB2 | 0.16 | 0.29 | 0.13 | 0.32 | 0.16 | 0.02 |
| FANCG | -0.08 | 0.15 | 0.23 | 0.11 | 0.19 | -0.04 |
| FANCF | 0.24 | 0.77 | 0.53 | 0.68 | 0.44 | -0.09 |
| FANCE | -0.03 | 0.59 | 0.62 | 0.64 | 0.66 | 0.04 |
| FANCC | 0.14 | -0.12 | -0.26 | -0.21 | -0.35 | -0.09 |
| FANCB | 0.23 | -0.38 | -0.61 | -0.34 | -0.57 | 0.04 |
| FANCA | -0.17 | 0.71 | 0.88 | 0.59 | 0.76 | -0.12 |
| BRIP1 | 0.1 | -0.81 | -0.91 | -1.03 | -1.14 | -0.22 |
| FANCM | 0.13 | 0.16 | 0.04 | 0.2 | 0.07 | 0.04 |
| FANCL | -0.07 | -0.05 | 0.01 | -0.22 | -0.16 | -0.17 |
| RAD51C | 0.14 | -0.56 | -0.7 | -0.59 | -0.72 | -0.02 |
| FANCI | 0.12 | 0.22 | 0.1 | 0.24 | 0.11 | 0.02 |
| SLX4 | 0.16 | -0.02 | -0.18 | 0.11 | -0.05 | 0.13 |
| RFWD3 | 0.09 | 0.60 | 0.51 | 0.63 | 0.54 | 0.03 |
| MAD2L2 | -0.08 | 0.58 | 0.66 | 0.52 | 0.60 | -0.06 |
| XRCC2 | 0.14 | 0.70 | 0.55 | 0.82 | 0.68 | 0.13 |
| UBE2T | -0.14 | 0.14 | 0.29 | 0.22 | 0.36 | 0.07 |
| ERCC4 | 0.14 | 0.31 | 0.17 | 0.33 | 0.19 | 0.02 |
| RAD51 | 0.20 | 0.18 | -0.02 | 0.06 | -0.14 | -0.12 |

**Table S6: Pairwise comparison of the number of exons in the most expressed transcript of genes in four Fanconi Anemia samples.** The fraction of expressed genes with varying exon counts when comparing within sample groups is 23.5% but the fraction of expressed genes with varying exon counts when comparing samples across sample groups is about 28.5%. This difference represents, on average, more than 750 genes with varying exon counts in their most expressed transcript.

| **Sample pair** | **Transcripts with equal exons** | **Expressed genes** | **Transcripts with varying exon count%** |
| --- | --- | --- | --- |
| FA1 - FA2 | 12221 | 15903 | 23 |
| FA_RV1 - FA_RV2 | 12016 | 15903 | 24 |
| FA1 - FA_RV1 | 11311 | 15903 | 29 |
| FA1 - FA_RV2 | 11351 | 15903 | 29 |
| FA2 - FA_RV1 | 11401 | 15903 | 28 |
| FA2 - FA_RV2 | 11432 | 15903 | 28 |

**Table S7: Ontological gene enrichment analysis of the 270 genes that were found to be differentially expressed the FA and FA_RV sample groups.** The exceptional FDR corrected p-values seen for these GO ontology terms, especially for terms involved in infectious disease, development and cancer illustrate the potential connections with the observed symptoms and diseases seen in FA patients. The DAVID online bioinformatics tool was used to perform this GO enrichment analysis.

| **Category** | **Term** | **Count** | **Genes** | **List Total** | **FDR** |
| --- | --- | --- | --- | --- | --- |
| BP | GO:0060337~type I interferon signaling pathway | 17 | IFITM1, IFITM3, OAS3, HLA-A, RSAD2, OAS1, HLA-B, OAS2, IFIT2, OASL, IFIT1, IFI27, ISG15, XAF1, MX1, MX2, IFI6 | 235 | 4.89E-13 |
| BP | GO:0030574~collagen catabolic process | 16 | COL4A2, COL4A1, MRC2, COL3A1, COL5A3, COL5A2, MMP2, MMP1, CTSL, CTSK, COL6A3, COL6A2, COL1A2, COL6A1, COL8A1, COL11A1 | 235 | 5.94E-12 |
| BP | GO:0009615~response to virus | 19 | IFIH1, CYP1A1, IFITM1, IFITM3, CLU, OAS3, RSAD2, OAS1, IFI44, OAS2, TRIM22, DDX58, IFIT2, OASL, IFIT1, MYD88, DDX60, MX1, MX2 | 235 | 6.35E-12 |
| BP | GO:0030198~extracellular matrix organization | 23 | COL4A2, COL4A1, COL3A1, FBN1, HSPG2, BCAN, OLFML2A, COL5A3, COL16A1, COL5A2, EMILIN1, LAMA4, ITGA5, LAMA5, FBLN5, COL6A3, TGFBI, COL1A2, COL6A2, COL6A1, MFAP2, COL8A1, COL11A1 | 235 | 1.67E-11 |
| BP | GO:0007155~cell adhesion | 28 | MTSS1, CCL2, FERMT1, BCAN, CDH4, LGALS3BP, FAP, TGFBI, COL6A3, COL6A2, COL6A1, CD24, GPNMB, COL8A1, ADAM23, COL16A1, MCAM, TPBG, GAS6, EMILIN1, LAMA4, PGM5, FREM2, ITGA5, CD33, SUSD5, SEMA4D, THEMIS2 | 235 | 8.26E-08 |
| BP | GO:0051607~defense response to virus | 17 | IFITM1, IFITM3, OAS3, RSAD2, APOBEC3G, IFI44L, OAS1, OAS2, TRIM22, IFIT2, OASL, IFIT1, ISG15, DDX60, MX1, MX2, GBP1 | 235 | 3.20E-07 |
| BP | GO:0045071~negative regulation of viral genome replication | 10 | IFIT1, OASL, ISG15, IFITM1, IFITM3, OAS3, RSAD2, APOBEC3G, OAS1, MX1 | 235 | 6.69E-07 |
| BP | GO:0071230~cellular response to amino acid stimulus | 8 | COL4A1, ASS1, COL3A1, COL1A2, COL6A1, COL16A1, MMP2, COL5A2 | 235 | 6.69E-04 |
| BP | GO:0035987~endodermal cell differentiation | 6 | COL4A2, ITGA5, COL6A1, COL8A1, COL11A1, MMP2 | 235 | 0.0051 |
| BP | GO:0060333~interferon-gamma-mediated signaling pathway | 8 | OASL, OAS3, HLA-A, OAS1, OAS2, HLA-B, TRIM22, GBP1 | 235 | 0.0085 |
| BP | GO:0022617~extracellular matrix disassembly | 8 | CTSL, CTSK, CAPG, FBN1, HSPG2, BCAN, MMP2, MMP1 | 235 | 0.0119 |
| BP | GO:0009612~response to mechanical stimulus | 7 | TXNIP, INHBB, CCL2, COL3A1, PPARG, BDKRB2, PIEZO2 | 235 | 0.0202 |
| BP | GO:0006955~immune response | 17 | SECTM1, CRIP1, CCL2, ENPP2, C3, IFITM3, OAS3, HLA-A, C1R, OAS1, OAS2, HLA-B, TRIM22, TNFRSF1B, TNFRSF10D, SEMA4D, IFI6 | 235 | 0.0317 |
| BP | GO:0001525~angiogenesis | 12 | COL4A2, CCL2, LAMA5, ITGA5, FAP, TGFBI, HSPG2, MCAM, COL8A1, TNFAIP2, MMP2, EPHB2 | 235 | 0.0313 |
| MF | GO:0005201~extracellular matrix structural constituent | 10 | COL4A2, LAMA4, COL4A1, COL3A1, FBN1, COL1A2, BCAN, COL5A3, COL11A1, COL5A2 | 233 | 0.0001 |
| MF | GO:0001730~2'-5'-oligoadenylate synthetase activity | 4 | OASL, OAS3, OAS1, OAS2 | 233 | 0.0021 |
| MF | GO:0005178~integrin binding | 10 | ADAM23, LAMA5, ITGA5, FAP, FBLN5, TGFBI, COL3A1, FBN1, GPNMB, COL16A1 | 233 | 0.0020 |
| MF | GO:0005518~collagen binding | 7 | CTSL, CTSK, C1QTNF1, TGFBI, MRC2, COL5A3, PCOLCE | 233 | 0.0172 |
| MF | GO:0003725~double-stranded RNA binding | 7 | DDX58, IFIH1, OASL, DDX60, OAS3, OAS1, OAS2 | 233 | 0.0151 |
| MF | GO:0016740~transferase activity | 8 | GSTM1, OASL, SPTLC3, XYLT1, GALNT5, OAS3, OAS1, OAS2 | 233 | 0.0241 |
| MF | GO:0048407~platelet-derived growth factor binding | 4 | COL4A1, COL3A1, COL1A2, COL6A1 | 233 | 0.0225 |
| MF | GO:0005509~calcium ion binding | 22 | S100A4, S100A6, ME3, LTBP1, ENPP2, CD248, PAMR1, FAM20C, FBN1, HSPG2, S100A11, S100A10, C1R, CDH4, MMP1, ANXA3, GAS6, FAT3, FBLN5, AIF1L, NINL, RCN3 | 233 | 0.0462 |

Count: The number of genes in the input list that was found to be associated with the given GO term. List total: The total number of genes in the input list that are associated with GO terms in the DAVID database.

**Table S8: Pathway analysis of 82 genes that were found to be associated with genomic variants that were specific to the FA sample group.** Pathway analysis also confirms the association of the differentially expressed genes with the observed signs, symptoms and diseases typically seen in FA patients. The pathway analysis was performed using the REACTOME pathway online tool.

| **Pathway name** | **#Entities found** | **#Entities total** | **FDR** |
| --- | --- | --- | --- |
| Translocation of ZAP-70 to Immunological synapse | 4 | 42 | 0.0364 |
| Phosphorylation of CD3 and TCR zeta chains | 4 | 45 | 0.0364 |
| PD-1 signaling | 4 | 45 | 0.0364 |

Entities found: Number of genes in the input list that were found to be associated with a pathway. Entities total: Total number of genes that are associated with a given pathway in the reactome database.

**Table S9: Pathway analysis of the 270 genes that were differentially expressed between the FA and FA_RV sample groups.**

| **Pathway name** | **Entities found** | **Entities total** | **Entities FDR** |
| --- | --- | --- | --- |
| Endosomal/Vacuolar pathway | 57 | 82 | 5.88E-15 |
| Antigen Presentation: Folding, assembly and peptide loading of class I MHC | 56 | 102 | 5.88E-15 |
| Degradation of the extracellular matrix | 33 | 148 | 5.88E-15 |
| Collagen degradation | 26 | 69 | 5.88E-15 |
| Class I MHC mediated antigen processing & presentation | 63 | 464 | 5.88E-15 |
| Antigen processing-Cross presentation | 59 | 186 | 5.88E-15 |
| ER-Phagosome pathway | 57 | 164 | 5.88E-15 |
| Interferon Signaling | 77 | 292 | 5.88E-15 |
| Immunoregulatory interactions between a Lymphoid and a non-Lymphoid cell | 63 | 316 | 5.88E-15 |
| Interferon alpha/beta signaling | 72 | 141 | 5.88E-15 |
| Cytokine Signaling in Immune system | 92 | 1006 | 5.88E-15 |
| Interferon gamma signaling | 62 | 176 | 5.88E-15 |
| Extracellular matrix organization | 40 | 329 | 2.72E-13 |
| Adaptive Immune System | 76 | 1086 | 9.59E-13 |
| Immune System | 129 | 2616 | 3.44E-11 |
| Assembly of collagen fibrils and other multimeric structures | 18 | 67 | 3.60E-11 |
| Activation of Matrix Metalloproteinases | 14 | 35 | 6.77E-11 |
| Collagen formation | 19 | 104 | 5.06E-09 |
| Collagen chain trimerization | 12 | 44 | 1.65E-07 |
| Integrin cell surface interactions | 14 | 87 | 5.53E-06 |
| Collagen biosynthesis and modifying enzymes | 13 | 76 | 7.45E-06 |
| ECM proteoglycans | 12 | 78 | 6.16E-05 |
| Non-integrin membrane-ECM interactions | 10 | 61 | 2.35E-04 |
| MET activates PTK2 signaling | 7 | 32 | 8.41E-04 |
| NCAM1 interactions | 8 | 44 | 8.41E-04 |
| MET promotes cell motility | 8 | 45 | 9.44E-04 |
| Laminin interactions | 5 | 31 | 0.0390 |
| Elastic fibre formation | 6 | 46 | 0.0390 |
| Antiviral mechanism by IFN-stimulated genes | 8 | 83 | 0.0459 |
| ISG15 antiviral mechanism | 8 | 83 | 0.0459 |

Entities found: Number of genes in the input list that were found to be associated with a pathway. Entities total: Total number of genes that are associated with a given pathway in the reactome database.
